# A Role for Nuclear Actin in HDAC 1 and 2 Regulation

**DOI:** 10.1038/srep28460

**Published:** 2016-06-27

**Authors:** Leonid A. Serebryannyy, Christina M. Cruz, Primal de Lanerolle

**Affiliations:** 1 Department of Physiology and Biophysics, University of Illinois at Chicago, IL, Chicago, USA

## Abstract

Class I histone deacetylases (HDACs) are known to remove acetyl groups from histone tails. This liberates positive charges on the histone tail and allows for tighter winding of DNA, preventing transcription factor binding and gene activation. Although the functions of HDAC proteins are becoming apparent both biochemically and clinically, how this class of proteins is regulated remains poorly understood. We identified a novel interaction between nuclear actin and HDAC 1 and HDAC 2. Nuclear actin has been previously shown to interact with a growing list of nuclear proteins including chromatin remodeling complexes, transcription factors and RNA polymerases. We find that monomeric actin is able to bind the class I HDAC complex. Furthermore, increasing the concentration of actin in HeLa nuclear extracts was able to suppress overall HDAC function. Conversely, polymerizing nuclear actin increased HDAC activity and decreased histone acetylation. Moreover, the interaction between class I HDACs and nuclear actin was found to be activity dependent. Together, our data suggest nuclear actin is able to regulate HDAC 1 and 2 activity.

Histone deacetylases (HDACs) are a family of proteins that remove acetyl groups from lysine residues^1^,^2^,^3^. Class I HDACs, in particular (HDAC 1, 2, 3 and 8), are found largely in the nucleus and are primarily responsible for the post-translational modification of histones into a deacetylated and more repressive state. As the acetyl group is removed from lysine residues on histone tails, histones become more basic and are able to tightly wrap around DNA. This epigenetic change generally restricts access to transcription machinery and alters nuclear signaling pathways involved in cell proliferation and survival^1^,^3^,^4^. Class I HDAC isoforms have been identified as components of multiple chromatin remodeling complexes essential for differential gene regulation^3^,^4^,^5^,^6^. Specifically, HDAC 1 and 2, which share 82% sequence homology, show a propensity to heterodimerize to perform their functions, yet exhibit independent activity in both a cell type and function dependent manner^5^. Indeed, HDACs have been implicated in a diverse range of functions, and HDAC inhibitors have been used for a variety of therapies targeting cancer, epilepsy, neurological disorders, immune disorders, parasitic infection, and cardiac dysfunction^5^,^7^. Still, relatively little is known about how different HDAC complexes maintain the transcriptome, let alone how they are regulated^3^,^7^.

Intriguingly, work by Joshi *et al*. have identified an interaction between HDAC 2 and actin related protein Arp4 (ACTL6, BAF53), implicating a novel function for nuclear actin; yet the physiological function of this interaction has not been ascertained^8^. Furthermore, a recent study showed that for specific inflammatory response genes the nuclear receptor co-repressor (NCoR) complex, which contains HDAC 3, is regulated by the actin binding activity of coronin 2A^9^. Nuclear fractionation studies have also suggested that HDAC proteins may alter their nuclear solubility in an actin dependent manner^10^. In addition, HDAC 8 has been shown to specifically interact with skeletal a-actin in the cytoplasm, potentially regulating cell contractility^11^. These studies point to multiple actin-dependent pathways by which HDAC function may be modulated.

Actin is canonically recognized as a component of the cytoskeleton and an important regulator of force production and movement in the cell^12^. However, a growing body of evidence has shown actin is also involved in many nuclear processes. Nuclear actin has been found to bind and facilitate transcription by all three RNA polymerases and multiple transcription factors^13^. Actin in the nucleus has also been identified as a cofactor in several nuclear signaling pathways and chromatin remodelers such as the INO80^14^,^15^, PCAF^16^, SWI/SNF^17^,^18^, P300/CBP^19^, p400^20^, Tip60^21^, NCoR^9^, and NuA4^18^,^22^ complexes. Although actin has been implicated in numerous nuclear functions^13^,^23^, how it performs these functions are poorly understood and require further study.

To identify new functions for nuclear actin, we performed mass spectrometry on pulldowns using purified actin coupled to Sepharose beads in HeLa nuclear extract. Our proteomics study identified a potential interaction between nuclear actin and HDAC 1 and 2. We confirmed this interaction by co-immunoprecipitation assays. Specifically, we found monomeric rather than polymerized actin to be the preferential binding partner of HDAC 1. We also found that this interaction was dependent on HDAC complex activity and could be altered following HL60 cell differentiation. The addition of purified actin to a nuclear extract led to a dose dependent inhibition of HDAC deacetylation activity. However, polymerization of nuclear actin increased HDAC activity and decreased histone 3 and histone 4 lysine 16 acetylation levels. Together our data suggest nuclear actin is able to bind the active HDAC 1 and 2 complex and attenuate its activity, potentially facilitating chromatin unwinding and gene transcription.

## Results

### Nuclear actin interacts with HDAC 1 and 2

**Figure 1:**
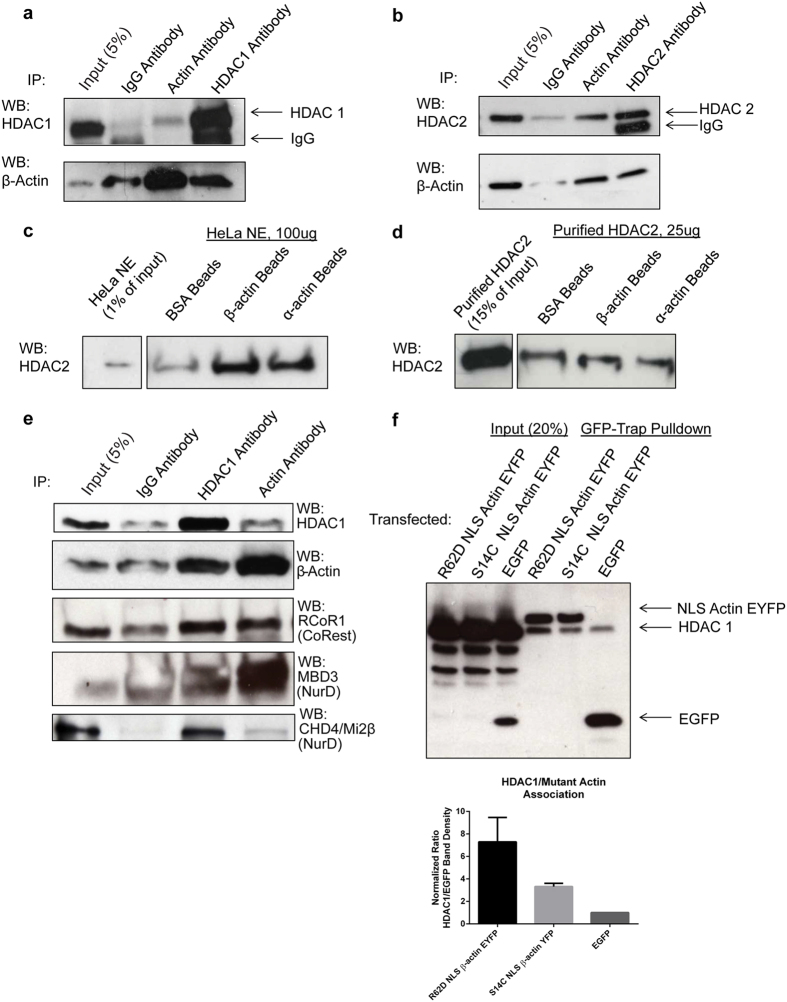
Nuclear actin associates with HDAC 1 and 2. (**a**) Co-immunoprecipitation of HeLa nuclear extract using non-specific IgG, HDAC 1, and β-actin antibodies and blotted for HDAC 1 and β-actin. (**b**) Co-immunoprecipitation of HeLa nuclear extract using non-specific IgG, HDAC 2, and β-actin antibodies and blotted for HDAC 2 and β-actin. (**c**) HeLa nuclear extract incubated with Sepharose beads coupled with purified BSA (control), β-actin, or α-actin. Pulldowns were performed and immunoblotted against HDAC 2 and β-actin. (**d**) Same as in (**c**) except beads were incubated with recombinant Flag-HDAC 2. Note the enrichment of HDAC 2 using actin beads is only present in the nuclear extract and not with purified HDAC 2 alone. (**e**) Co-immunoprecipitation of crosslinked HeLa nuclear extract using non-specific IgG, HDAC 1, and β-actin antibodies and blotted for the listed proteins. (**f**) GFP pulldown assay on crosslinked HeLa cells transfected with GFP or NLS β-actin YFP with the S14C (polymerization promoting) or R62D (polymerization resistant) mutation. Blots were probed for GFP and HDAC 1. Note the increased presence of HDAC 1 in the R62D NLS β-actin YFP precipitated lane. Data were quantified and HDAC1 band density was normalized to GFP band density. Mean + SEM, N = 3, 1-way ANOVA.

To explore the role of actin in the nucleus, we performed pulldown assays in HeLa nuclear extract using purified non-muscle actin covalently coupled to Sepharose beads or bovine serum albumin (BSA) coupled beads as a control. Mass spectrometry on the eluted fractions revealed multiple nuclear actin binding partners implicated in histone remodeling (Table S1, Fig. S1a). Among the strongest candidates were HDAC 1 and 2. To confirm that nuclear actin interacts with HDAC 1 and 2, we performed co-immunoprecipitation experiments on HeLa nuclear extract using IgG as a control and antibodies to actin, HDAC 1, or HDAC 2 (Fig. 1a,b, quantified in Fig. S1b). Western blots probed with antibodies to actin, HDAC 1 and 2 revealed an association between actin and HDAC 1 and 2. To assess if this was a direct or indirect protein-protein interaction, we covalently coupled purified non-muscle actin, skeletal α-actin, or BSA, as a control, to Sepharose beads. These beads were then incubated in HeLa nuclear extract (Fig. 1c) or with purified HDAC 2 (Fig. 1d), precipitated, and probed with HDAC 2 antibody. Both α- and non-muscle actin bound Sepharose brought down more HDAC 2 than BSA Sepharose beads (Fig. 1c) in HeLa nuclear extract, confirming our antibody co-immunoprecipitation experiments (Fig. 1b). However actin beads incubated with only purified HDAC 2 did not show any enrichment (Fig. 1d), suggesting nuclear actin most likely binds to a component of the class I HDAC complex rather than directly to the HDAC proteins.

To determine potential intermediates between nuclear actin and HDAC 1 and 2, we analyzed our actin pulldown mass spectrometry data to identify reported HDAC binding partners and confirmed these interactions using co-immunoprecipitation experiments 1e and S1a. Although we were unable to conclude which proteins mediate the actin/HDAC 1 and 2 interaction, co-immunoprecipitation assays with actin antibodies revealed an interaction with proteins found in both the CoREST and NuRD chromatin remodeling complexes (Fig. 1e). Thus, our results suggest actin and class I HDACs may be components of multiple chromatin remodeling complexes including those already published such as the NCoR complex^3^,^9^,^23^, as well as the CoREST and NuRD complexes identified here.

Because of actin’s unique ability to exist as monomers or polymers of different lengths, we assessed if nuclear actin preferentially binds the HDAC complex in its monomeric or polymeric form. HeLa cells were transfected with constructs encoding a polymerization resistant (R62D) or a polymerization promoting (S14C) actin mutant^24^ coupled to a nuclear localization signal (NLS) and EYFP (Fig. 1f). Cells transfected with R62D or S14C NLS β-actin EYFP or EGFP as a control were reversibly crosslinked, lysed and precipitated with GFP-Trap beads. Immunoblotting for HDAC 1 and GFP showed an enrichment of HDAC 1 in the R62D NLS β-actin EYFP pulldown despite similar levels of precipitated GFP tagged proteins. This suggests that the HDAC complex preferentially binds non-polymerized actin, which is in agreement with recent studies showing monomeric actin bound to other chromatin remodeling complexes such as INO80, NuA4, and SWI/SNF via their helicase-SANT–associated (HSA) domain^18^.

### The interaction of nuclear actin and HDAC 1 and 2 is activity dependent

**Figure 2:**
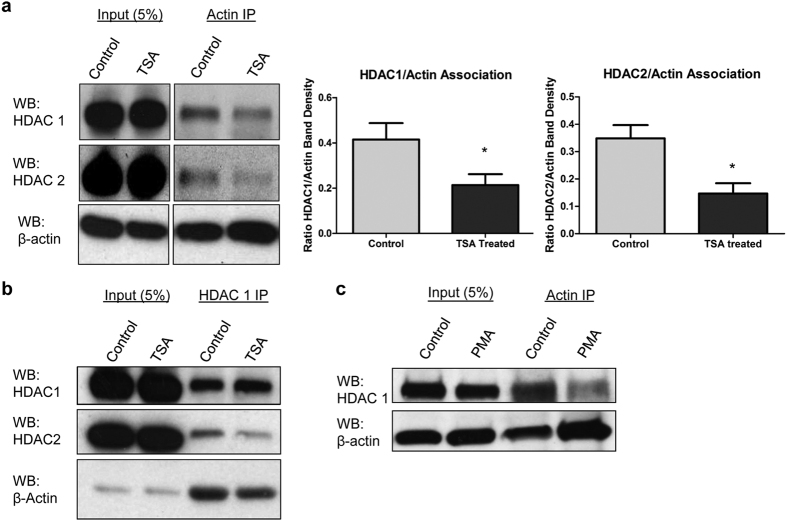
The nuclear actin/HDAC 1 and 2 interaction is activity dependent. (**a**) HeLa cells were treated with 4 μM TSA for 3 h and crosslinked with DSP before extraction. Extracts were prepared and immunoprecipitated with β-actin antibody and immunoblotted for β-actin, HDAC 1 and 2. Band densities were quantified in the β-actin IP lane and calculated as a ratio of HDAC 1 intensity to β-actin. Note the decrease in the association between β-actin and HDAC 1 and HDAC2 in the TSA treated cells vs. control. Mean + SEM, N = 5, *p < 0.05 by t-test. (**b**) Same as in (**a**) except extracts were immunoprecipitated with HDAC 1 antibodies and immunoblotted for β-actin, HDAC 1, and HDAC 2. Note that TSA decreases not only the interaction between β-actin and HDAC 1 and 2 as in (**a**), but also the interaction between HDAC 1 and HDAC 2, suggesting the entire HDAC complex is dissociated. (**c**) HL60 cells were left untreated (control) or treated with 50 ng/mL PMA for 72 h to induce a differentiated monocytic phenotype. Cells were then lysed, immunoprecipitated with antibodies to β-actin, then blotted with antibodies to β-actin and HDAC 1.

To test if the interaction between nuclear actin and the HDAC 1 and 2 complex is dynamically regulated, we treated HeLa cells with vehicle or the HDAC inhibitor, trichostatin A (TSA), before extraction. Cells were then reversibly crosslinked to maintain their protein-protein interactions, lysed, and precipitated as in Fig. 1. Quantification of co-immunoprecipitation experiments showed a ~50% decrease in the amount of nuclear actin bound to both HDAC 1 and 2 following TSA treatment (Figs 2a and S1c). Furthermore, HDAC 1 immunoprecipitation experiments show that treatment with TSA decreases the interaction between HDAC 1 and HDAC 2, as well as the interaction with actin (Fig. 2b). These experiments suggest nuclear actin binds to the active HDAC complex and the inhibition/dissociation of the complex impairs nuclear actin binding.

We further evaluated the interaction between nuclear actin and HDAC 1 using a model of cell differentiation. HL60 cells are a human promyelocytic leukemia cell line that can differentiate into a monocytic phenotype when treated with phorbol-12-myristate-13-acetate (PMA)^25^,^26^. Differentiation of HL60 cells has been shown to alter the interaction between nuclear HDACs and their associating protein complexes^26^,^27^. It has been shown that nuclear actin may also be a determinant in the differentiation of HL60 cells^28^. Therefore, we treated HL60 cells with PMA for 72 h and performed immunoprecipitation experiments using antibodies to β-actin and probed for changes in associating levels of HDAC 1 (Fig. 2c). This experiment showed less HDAC1 in the actin immunoprecipitate of differentiated cells compared to non-differentiated HL60 cells. These experiments suggest nuclear actin is able to preferentially bind the HDAC complex in an activity dependent manner.

### Nuclear actin inhibits Class I HDAC activity

**Figure 3:**
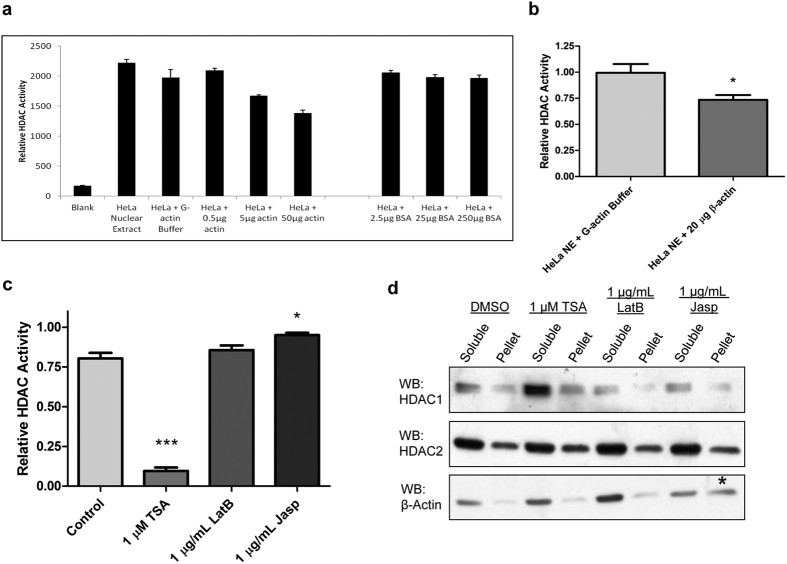
Nuclear actin regulates class I HDAC activity *in vitro*. (**a**) HDAC activity of HeLa nuclear extracts incubated with purified non-muscle actin or purified bovine serum albumin (BSA) in G-actin buffer. Mean + SD, done in triplicate. (**b**) Average HDAC activity in HeLa nuclear extracts after the addition of buffer or 20 μg of purified non-muscle actin. Data were normalized to non-treated HeLa nuclear extract. Mean + SEM, N = 6, *p < 0.05 by t-test. (**c**) HDAC activity of HeLa nuclear extract incubated with DMSO (Control), TSA (HDAC inhibitor), latrunculin B (LatB, actin depolymerizing), or jasplakinolide (Jasp, actin polymerizing). Data were normalized to non-treated HeLa nuclear extract. Mean + SEM, N ≥ 3, 1-way ANOVA. (**d**) Sedimentation assay of HeLa nuclear extract treated as in (**c**) and centrifuged 100,000 × g for 1 h, and blotted for β-actin, HDAC 1, and HDAC 2. Note the increase in polymerized nuclear actin (pelleted actin) with jasplakinolide treatment (asterisks).

We next assessed if nuclear actin is able to modulate HDAC activity. To do so, lysine deacetylase activity was measured using an *in vitro* fluorometric assay^29^. HeLa nuclear extract was incubated with purified non-muscle actin or BSA as a control, synthetic HDAC substrate was added, and HDAC activity was assayed as a function of substrate deacetylation. Nuclear extract incubated with increasing amounts of purified actin showed a dose dependent inhibition of class I HDAC activity, yet nuclear extract incubated with 5-fold more BSA showed no effect (Fig. 3a). Indeed, we found a significant decrease in HDAC activity in nuclear extracts incubated with 20 μg of purified actin over several separate experiments (Fig. 3b). In agreement with the pulldown assays using purified HDAC 2 and actin (Fig. 1d), incubation of purified HDAC 2 and actin had no effect on activity, further suggesting that actin regulates HDAC activity indirectly (Fig. S2a). Although actin has previously been reported to be acetylated^30^,^31^, we found no change in actin acetylation levels when cells were treated with TSA or when purified actin was incubated with HDAC 2 (Fig. S2b), further eliminating the possibility that actin was serving as a competitive substrate.

Given the preferential interaction of HDAC 1 with monomeric actin (Fig. 1f), we predicted that polymerizing actin may sequester actin monomers and increase HDAC activity. Therefore, we pre-treated HeLa nuclear extract with either TSA as a positive control, latrunculin B to depolymerize actin, or jasplakinolide to increase actin polymerization before assaying HDAC activity (Fig. 3c,d). Because most of the actin within the nucleus is already soluble after sedimentation, pre-treatment with latrunculin B resulted in only a modest increase in soluble nuclear actin levels (Fig. 3d) and a negligible change in HDAC activity (Fig. 3c). In contrast, pre-treatment of nuclear extracts with jasplakinolide showed an increase in actin polymerization, a corresponding decrease in soluble actin levels as determined by sedimentation assay (Fig. 3d), and a small but significant increase in HDAC activity (Fig. 3c).

**Figure 4:**
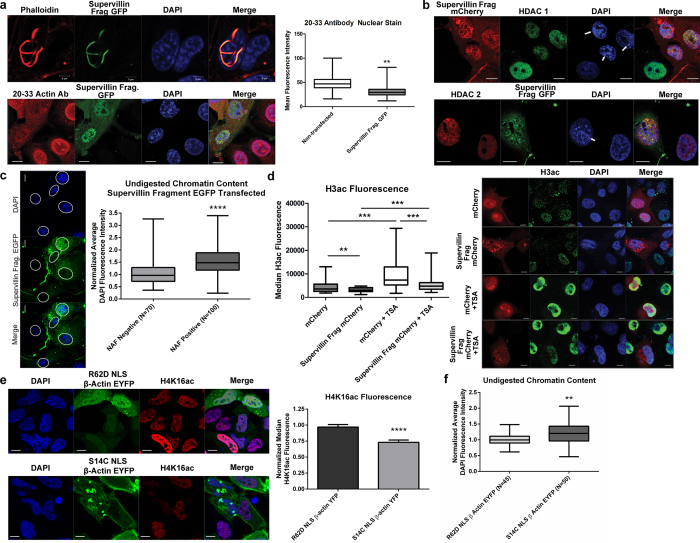
Nuclear actin polymerization regulates class I HDAC activity in culture. (**a**) Cells transfected with a fragment of the supervillin actin bundling protein (green) were fixed and stained with phalloidin (red, top) to mark polymerized endogenous nuclear actin or actin antibody (red, bottom) to label nuclear actin monomers. The fluorescence intensity of the antibody staining, a measure of the level of free actin, was quantified (N ≥ 16, **p < 0.01 by t-test). Note the formation of phalloidin labeled nuclear actin filaments leads to decreased levels of monomeric actin in the nucleus. (**b**) HeLa cells were stained with antibodies to HDAC 1 (top, green) or HDAC 2 (bottom, red). Note the altered HDAC distribution and increased chromatin foci in cells with nuclear filaments (arrows), presumably because monomeric actin is sequestered into nuclear filaments. Scale bars = 10 μm. (**c**) Demonstrative micrograph of COS7 cells transfected with supervillin fragment EGFP (green), fixed in methanol, digested with DNase I and stained with DAPI (blue). Average DAPI fluorescence of the entire nucleus was quantified in cells with nuclear actin filaments (NAF positive) and EGFP positive cells with only cytoplasmic fluorescence (NAF negative). Values were normalized to DAPI fluorescence levels of EGFP negative cells in each field of view. Note the presence of increased DAPI fluorescence in cells with nuclear actin filaments formed by supervillin fragment EGFP expression, indicating increased heterochromatin content. Min/max box plots are shown. ****p < 0.0001 by t-test. Nuclei are marked by dotted lines. Scale bars: 10 μm. (**d**) HeLa cells transfected with mCherry or mCherry supervillin fragment (red) and left untreated or treated with 4 μM TSA for 3 h, fixed, and stained for acetylated histone 3 (H3ac, green). N ≥ 29, **p < 0.01 and ***p < 0.001 by ANOVA. Box plot shows 1–99% percentile. Note the decrease in H3ac fluorescence in nuclei with actin filaments and the increase in activity with TSA treatment. (**e**) HeLa cells transfected with R62D or S14C NLS β-actin EYFP (green) for 48 h were fixed and stained for acetylated H4K16 (red). Median fluorescence intensity was normalized to non-transfected cells in each frame. Images were acquired from 3 independent experiments. N = 105 cells and 140 cells, respectively. Mean + SEM. ****p < 0.0001 by t-test. (**f**) COS7 cells transfected with R62D or S14C NLS β-actin EYFP for 48 h were fixed in methanol, digested with DNase I and stained with DAPI. Heterochromatin content was assessed by measuring DAPI fluorescence intensity of the entire nucleus. The mean fluorescence intensity of nuclear actin EYFP expressing cells was normalized to EYFP negative cells in each frame. Min/max box plots are shown. Demonstrative images are shown in Fig. S3c. ** p < 0.01 by t-test.

To assess the effects of nuclear actin polymerization in cells, we induced the formation of nuclear actin filaments by expressing the N-terminus fragment of supervillin, an actin bundling protein that contains multiple nuclear localization signals and induces the polymerization of nuclear actin^32^. Phalloidin staining is a specific marker for actin filaments, whereas most actin antibodies, including the 20–23 actin antibody used in this study, stain depolymerized actin. Thus, phalloidin staining can be used to identify nuclei with actin filaments and antibody staining can be used to quantify the level of unpolymerized actin in the nucleus. Indeed, supervillin transfected cells showed decreased nuclear monomeric actin levels by immunolabeling, further indicating that the formation of nuclear actin filaments sequesters nuclear actin monomers (Fig. 4a).

Nuclei with actin filaments formed by expressing supervillin fragment mCherry showed an altered distribution of HDAC 1 and 2 and chromatin as compared to non-transfected cells (Fig. 4b). In agreement with the GFP pulldowns (Fig. 1f), neither HDAC 1 nor 2 co-localized with the polymerized actin in nuclear actin filaments (Figs. 4b and S3a). To assess levels of chromatin repression, we performed DNase I I digestion, which more readily digests open chromatin, on fixed cells and quantified the remaining DAPI fluorescence as a measure of heterochromatin content (Fig. 4c)^33^,^34^. We find cells with nuclear actin filaments induced by supervillin fragment EGFP expression contain significantly more DNase I resistant chromatin. To determine if HDAC activity was altered in nuclei with actin filaments, cells were transfected, stained for histone 3 acetylation before and after TSA treatment, and median fluorescence intensity was quantified (Fig. 4d). Nuclei with actin filaments displayed decreases in histone 3 acetylation consistent with a more active HDAC complex, as would be expected if less actin was bound to the HDAC complex. Although both nuclei with and without filaments had significantly higher levels of acetylation after TSA treatment, acetylation levels in the mCherry supervillin fragment + TSA group were still significantly lower than the mCherry + TSA control, suggesting a partial rescue of activity with TSA treatment. Furthermore, we transfected HeLa cells with R62D NLS β-actin EYFP or S14C NLS β-actin EYFP and examined histone 4 lysine 16 acetylation levels (H4K16ac), a specific and direct target of HDAC 1 and 2 (Figs. 4e and S3)^35^. We find that inducing nuclear actin polymerization with the S14C NLS β-actin construct coincided with decreased H4K16ac levels and increased condensation of chromatin as measured by DNAse I digestion (Figs. 4f and S3c). Presumably, this is due to lowered levels of nuclear monomeric actin and increased HDAC activity, consistent with the effects of jasplakinolide treatment (Fig. 3c,d) and supervillin expression (Fig. 4c,d).

## Discussion

Nuclear HDACs have been shown to be critical for maintaining the cell’s genetic program^6^. Indeed, studies have demonstrated HDAC inhibition to be a promising therapy for a wide range of pathologies^5^,^7^,^36^. HDAC 1 and 2 are largely found as components of at least three major protein complexes: Sin3, NuRD, and CoREST complexes^3^,^4^,^5^. Nonetheless, the full composition of these complexes, how these complexes are regulated, and how they differentially coordinate epigenetic changes in the cell remain outstanding questions. Our data identify nuclear actin as a novel binding partner of HDAC 1 and 2 (Fig. 1). Furthermore, this interaction is functional, since addition of purified actin to nuclear extract was able to suppress class I HDAC activity in a dose dependent manner (Fig. 3), and inhibiting the HDAC complex in culture reduced the HDAC/actin interaction (Fig. 2).

Although our results demonstrate actin is an integral regulator of the HDAC 1 and 2 complex, this interaction likely occurs through intermediary proteins and/or the NuRD and CoREST remodeling complexes (Figs. 1e and S1a, Table S1). Indeed, previous studies have implicated multiple roles for actin binding proteins within these complexes. APC, a WNT pathway mediated actin binding protein, is able to bind the CoREST complex through its interaction with CtBP^37^,^38^. Nuclear p120 catenin, another actin regulatory protein, has also been found to bind the CoREST complex, displace it from DNA, and regulate differentiation^39^. Furthermore, the transcriptional co-repressor NAC1 is able to bind both monomeric actin^40^ and CoREST using its BTB domain^41^. In support, our proteomics experiment has implicated several key components of class I HDAC complexes, including NuRD and CoREST, as putative actin binding proteins (Figs. 1e and S1a, Table S1). These findings agree with a growing number of studies implicating nuclear actin in chromatin remodeling and transcription^13^,^23^, although it remains largely unclear why actin binding proteins are recruited to these complexes. Intriguingly, the nuclear localization and activity of multiple actin regulatory proteins have been found to be influenced by their acetylation state, including cortactin^42^, Net1A^43^, c-Abl^44^, zyxin^45^ and potentially many others^46^,^47^. These studies implicate a close connection between the regulation of protein acetylation, nuclear localization, and actin dynamics.

HDACs have been shown to complex with HSA domain proteins. This domain is found in actin binding chromatin remodelers such as the SWI/SNF, INO80, and RSC complexes^3^,^18^,^23^. In agreement with our pulldowns, crystal structures and biochemical assays have suggested that actin bound in the helicase SANT associated domain is likely monomeric^14^,^18^,^48^. However, evidence also exists that some of these complexes can bind actin filaments^10^,^49^. Therefore, how the polymerization and binding of nuclear actin affects chromatin remodeling remains a major question.

Previous work on inflammatory gene activation showed that the actin regulatory protein, coronin 2A, is an essential component of the NCoR-HDAC 3 complex^9^. This study also discovered that nuclear actin is recruited upon inflammatory gene induction and is necessary for NCoR clearance using the 2G2 actin antibody, while treatment with latrunculin A could partially prevent NCoR clearance. The recruitment of actin to NCoR was found to depend on coronin 2A’s actin binding domain. Therefore, the authors suggested that coronin’s interaction with oligomers of nuclear actin may lead to the release of the NCoR-HDAC 3 complex. Our results similarly show that transient recruitment of actin to the HDAC 1 and 2 complex inhibits its activity (Fig. 3). However in the case of HDAC 1 and 2, we find that bound nuclear actin is likely monomeric and binding occurs after transcriptional activation, since less actin was bound to the inhibited HDAC complex (Fig. 2a). Together, these studies suggest actin is a critical mediator of class I HDAC function, and although the mechanism remains unclear, the local polymerization and depolymerization of nuclear actin may be an elegant epigenetic regulator.

Polymerized actin has been implicated in several roles within the nucleus including the regulation of Oct4 in transplanted oocytes^50^ and the MAL/SRF pathway^51^,^52^. However, imaging studies using a probe specific to polymerized actin derived from a fragment of the utrophin protein have shown that polymerized actin does not co-localize with sites of transcription (RNAPI, II, III, splicesomes) nor chromatin remodeling complexes (Brg1, SWR1, coronin2A and BAF53/Arp4), unlike monomeric actin^53^. Indeed, we find that polymerizing nuclear actin, which is predicted to decrease the number of actin monomers, stimulates HDAC activity *in vitro* (Fig. 3c,d). Increasing nuclear actin polymerization in culture corresponds with increased chromatin compaction and decreased histone 3 acetylation as well as histone 4 lysine 16 acetylation levels (Figs. 4 and S3). Moreover, HDAC 1 and 2 do not co-localize with polymerized nuclear actin filaments (Fig. 4a), in agreement with our pulldown data (Fig. 1f). Although changes in HDAC activity or transcription could affect HDAC protein levels downstream, we did not note significant changes in HDAC protein levels (Figs. 4b,e and S3). This further suggests, along with our *in vitro* assays (Fig. 3), that actin inhibits HDAC activity.

In conclusion, nuclear actin has been shown to bind a wide range of nuclear complexes. Our study contributes to the understanding of how nuclear actin regulates gene expression and specifies one of a few reported instances where nuclear actin may work as an inhibitor. Our data suggest a model whereby nuclear actin is able to transiently bind the active HDAC 1 and 2 complex and attenuate its activity. When HDAC activity is inhibited, actin bound to HATs and chromatin remodelers would be able to decondense chromatin and recruit the RNA polymerase/actin complex to facilitate transcription.

## Materials and Methods

### Cell Culture and Antibodies

HeLa and COS7 cells were obtained from American Type Culture Collection and cultured in Dulbecco’s Modified Eagle’s Medium (Corning) supplemented with 1% penicillin/streptomycin (Invitrogen) and 10% fetal bovine serum (Invitrogen). Cells were incubated at 37 °C and 5% CO_2_. Cell transfections were carried out using Polyjet (SignaGen). HL60 cells were a kind gift from Dr. David Ucker (University of Illinois at Chicago). HL60 cells were cultured in RPMI with 10% fetal bovine serum and 1% penicillin/streptomycin. To differentiate HL60 cells, 50 ng/mL PMA (Santa-Cruz) was added directly to the media for 72 h until the cells obtained an adherent phenotype. Latrunculin B (Enzo Life Sciences), Jasplakinolide (EMD Millipore), and TSA (Santa-Cruz) were used as indicated.

Antibodies used include mouse monoclonals to HDAC 1, HDAC 2, GFP (Abcam), actin (Ac-15, Sigma-Aldrich), non-specific IgG (Santa-Cruz), Myc (9E10, Santa-Cruz), and acetyl lysine (Cytoskeleton Inc.).

Polyclonal antibodies used were HDAC 1, HDAC 2, and GFP (Abcam), acetyl histone 3 (Santa-Cruz), RCOR1, MBD3, CHD4, histone 3 (Bethyl), actin (20–33, Sigma Aldrich). Secondary antibodies used were Dylight 488- (Thermo Scientific), and Cy3- (Jackson Labs) conjugated goat IgGs, as well as fluorescein labeled rabbit anti-goat antibody (Jackson Labs). Mounting media containing DAPI (Vectashield) was used for ICC. Rhodamine conjugated phalloidin (Cytoskeleton Inc.) was used to stain for nuclear actin filaments. Primary antibody binding in western blots was detected with HRP conjugated secondary antibodies (Jackson Labs) using ECL reagent (Denville).

### Sepharose Bead Pulldowns

150 mg of Sepharose beads were coupled to 1 mg of purified non-muscle actin, rabbit skeletal actin (Cytoskeleton Inc) or purified BSA (Sigma-Aldrich) in coupling buffer (10 mM NaHCO_3_, 0.2 mM CaCl_2_, 0.2 mM ATP). Beads were then equilibrated in transcription buffer (20 mM HEPES pH 7.9, 20% glycerol, 100 mM KCl, 0.2 mM EDTA, 0.5 mM PMSF, and 0.5 mM DTT).

Bead pulldowns were performed by incubating protein coupled beads overnight at 4 °C with either 100 μg of HeLa nuclear extract (Promega) or 25 μg purified FLAG-HDAC 2 (BPS Bioscience). Beads were then washed 5 times in transcription buffer and once in 5X Tris-buffered saline (250 mM Tris-HCl pH 7.5 and 750 mM NaCl). Proteins were eluted from the beads using 8 M urea buffer (8 M urea, 1 mM DTT, 10 mM Tris-HCl pH 7.5). Proteomics and informatics services were provided by the CBC-UIC Research Resources Center Mass spectrometry, Metabolomics and Proteomics Facility which was established in part by a grant from The Searle Funds at the Chicago Community Trust to the Chicago Biomedical Consortium. Mass spectrometry results were sorted in Scaffold viewer by protein identification probability.

### Immunostaining and Microscopy

Cells were plated on glass coverslips at least 24 h before transfection. After transfection, cells were fixed in 4% PFA for 10 m then permeabilized with 0.3% Triton X100 (Sigma-Aldrich). Cells were then washed with PBS and incubated in 2% BSA for 1 h. Cells were stained using a humidity chamber. Primary antibody was added for 1 h at room temperature or overnight at 4 °C. Secondary antibody was added for 1 h at room temperature. Cells were mounted using Vectashield containing DAPI. Confocal images were obtained using a Zeiss LSM 710 microscope and image analysis was performed using Image J.

### Immunoprecipitation Assays

HeLa cells were pre-treated as indicated then collected in PBS and lysed in 10 volumes of 50 mM Tris-HCl at pH 7.5, 2 mM EDTA, 150 mM NaCl and 1% Triton X-100 (IP Buffer) followed by brief sonication. Where indicated, cells were first chemically crosslinked in 1 mM DSP (Dithiobis [succinimidyl propionate], Thermo Scientific) before lysis. For nuclear specific immunoprecipitation assays, HeLa cells were first lysed in hypotonic buffer (10 mM HEPES-KOH pH 7.9, 10 mM KCl, 1.5 mM MgCl_2_, 0.5 mM DTT, 0.5 mM PMSF) with mechanical perturbation. Nuclei were separated by centrifugation and lysed to make nuclear extract. HeLa extract was then incubated with the indicated antibodies overnight at 4 °C. Protein G magnetic beads (20 μl of a 50% solution; Thermo Scientific) were added and the mixture was incubated for another 2 h at 4 °C. The beads were washed extensively in IP buffer, followed by a wash in 1X TBS, and eluted by boiling in SDS sample buffer. In the GFP pulldowns, either GFP antibody or GFP-Trap magnetic beads (20 μl of a 50% solution; Chromotek) were used.

### *In vitro* HDAC Activity Assay

HDAC activity assays were performed in 96-well opaque microplates as previously described^29^. 20 μg of HeLa nuclear extract (Promega) or 50 ng recombinant Flag-HDAC 2 (BPS Bioscience) were diluted in assay buffer 1 (25 mM Tris-HCl, pH 8.0, 137 mM NaCl, 2.7 mM KCl, 1 mM MgCl_2_, and 1 mg/mL BSA) and incubated with the indicated amounts of pharmacological compounds, purified actin (Cytoskeleton Inc.) or BSA (Sigma-Aldrich) for 15 m at room temperature. Boc-L-Lys (AC)-AMC substrate in assay buffer 2 (25 mM Tris-HCl, pH 8.0, 137 mM NaCl, 2.7 mM KCl, 1 mM MgCl_2_) diluted to 25 μM was then added for 1 h. The reaction was stopped with 1 mg/mL trypsin (Sigma-Aldrich) and 5 μM trichostatin A (Santa-Cruz) in assay buffer 2. Plates were read using an excitation wavelength of 360 nm and an emission wavelength 460 nm.

### DNase I Digestion

To determine heterochromatin content, transfected COS7 cells were fixed in cold methanol then treated with 0 μg/mL of DNase I (Thermo-scientific) for 2 h at 37 °C, stained with DAPI, and imaged. Chromatin that is an open confirmation or areas of gene activity (euchromatin) should be more readily digested than areas of closed chromatin (heterochromatin)^33^,^34^. Chromatin content was measured as a function of mean DAPI stain fluorescence intensity of the entire nucleus.

### Sedimentation Assay

HeLa cells were extracted in hypotonic buffer (10 mM HEPES pH 7.9, 1.5 mM MgCl_2_, 10 mM KCl, 0.5 mM DTT) followed by mechanical perturbation through a 25 g needle to isolate nuclei and centrifuged for 10 min at 10,000 RPM. Nuclei were then lysed in transcription buffer (20 mM HEPES pH 7.9, 20% glycerol, 100 mM KCl, 0.2 mM EDTA, 0.5 mM PMSF, and 0.5 mM DTT) with a 30 g needle and cleared by centrifugation. HeLa nuclear extracts were spun at 100,000 × g for 1 h at 10 °C to pellet nuclear actin. Soluble fractions were directly boiled in hot SDS, pellet fractions were suspended in hot SDS then sonicated to ensure complete resuspension.

## Additional Information

**How to cite this article**: Serebryannyy, L. A. *et al*. A Role for Nuclear Actin in HDAC 1 and 2 Regulation. *Sci. Rep.* **6**, 28460; doi: 10.1038/srep28460 (2016).

## Supplementary Material

Supplementary Information
