# Supplementary material for: A Role for Nuclear Actin in HDAC 1 and 2 Regulation
Source: Sci Rep. 2016 Jun 27;6:28460. doi: 10.1038/srep28460 (PMC4921920; doi:10.1038/srep28460)
Supplement: Supplementary Information [file srep28460-s1.pdf]

A Role for Nuclear Actin in HDAC 1 and 2 Regulation

Leonid A. Serebryannyy, Christina M. Cruz and Primal de Lanerolle

From the Department of Physiology and Biophysics, University of Illinois at Chicago, Chicago, IL

SUPPLEMENTARY INFORMATION:

SUPPLEMENTARY FIGURE LEGENDS

Figure S1: Predicted binding partners of nuclear actin and HDAC 1 and 2. (**a**) Canonical protein composition of the common HDAC 1 and 2 chromatin regulatory complexes. Proteins that were identified by proteomics analysis of actin pulldowns are in bold faced black text on grey background. Unidentified proteins are in grey text on a white background. (**b**) Quantification of co-immunoprecipitation experiments as performed in **Figures 1a and b**. (**c**) Co-immunoprecipitation of DSP crosslinked HeLa nuclear extract using non-specific IgG, HDAC 1, and β-actin antibodies and blotted for HDAC 1 and β-actin. Protein-antibody bound beads were extracted in SDS buffer with 2-mercaptoethanol to reduce crosslinks before gel loading.

Figure S2: Nuclear actin alone does not directly inhibit HDAC 2 nor act as an HDAC 2 substrate. (**a**) HDAC activity of recombinant Flag-HDAC 2, purified non-muscle actin, substrate alone (blank), or all three incubated in G-actin or transcription buffer. Mean + SD, done in triplicate. (**b**) HeLa cells treated with 4 μM TSA for 3 h, LatB 1 μM 1h, or vehicle were extracted, immunoprecipitated with β-actin antibody, and immunoblotted for acetylated lysine residues. Similarly 50 μg of purified non-muscle actin was incubated with and without 7 μg of recombinant Flag-HDAC 2 and immunoblotted (right 2 lanes). No treatment showed visible changes in acetylation of the predicted actin band.

Figure S3: S14C NLS β-actin EYFP expression affects H4K16ac levels. (**a**) HeLa cells transfected with S14C NLS β-actin EYFP or R62D NLS β-actin EYFP and stained for HDAC1. (**b**) HeLa cells transfected with S14C NLS β-actin EYFP or R62D NLS β-actin EYFP or GFP as a control and blotted for the indicated proteins. (**c**) Representative images from **Figure 4f**. COS7 cells transfected with S14C NLS β-actin EYFP or R62D NLS β-actin EYFP (green) were fixed in methanol, digested with DNase I, and stained with DAPI (blue).

Table S1: Mass spectrometry results from actin-Sepharose bead pulldown and BSA-Sepharose bead pulldown. Protein identifications that appeared in the BSA-Sepharose pulldown were crossed out.

Supplementary Figure S1

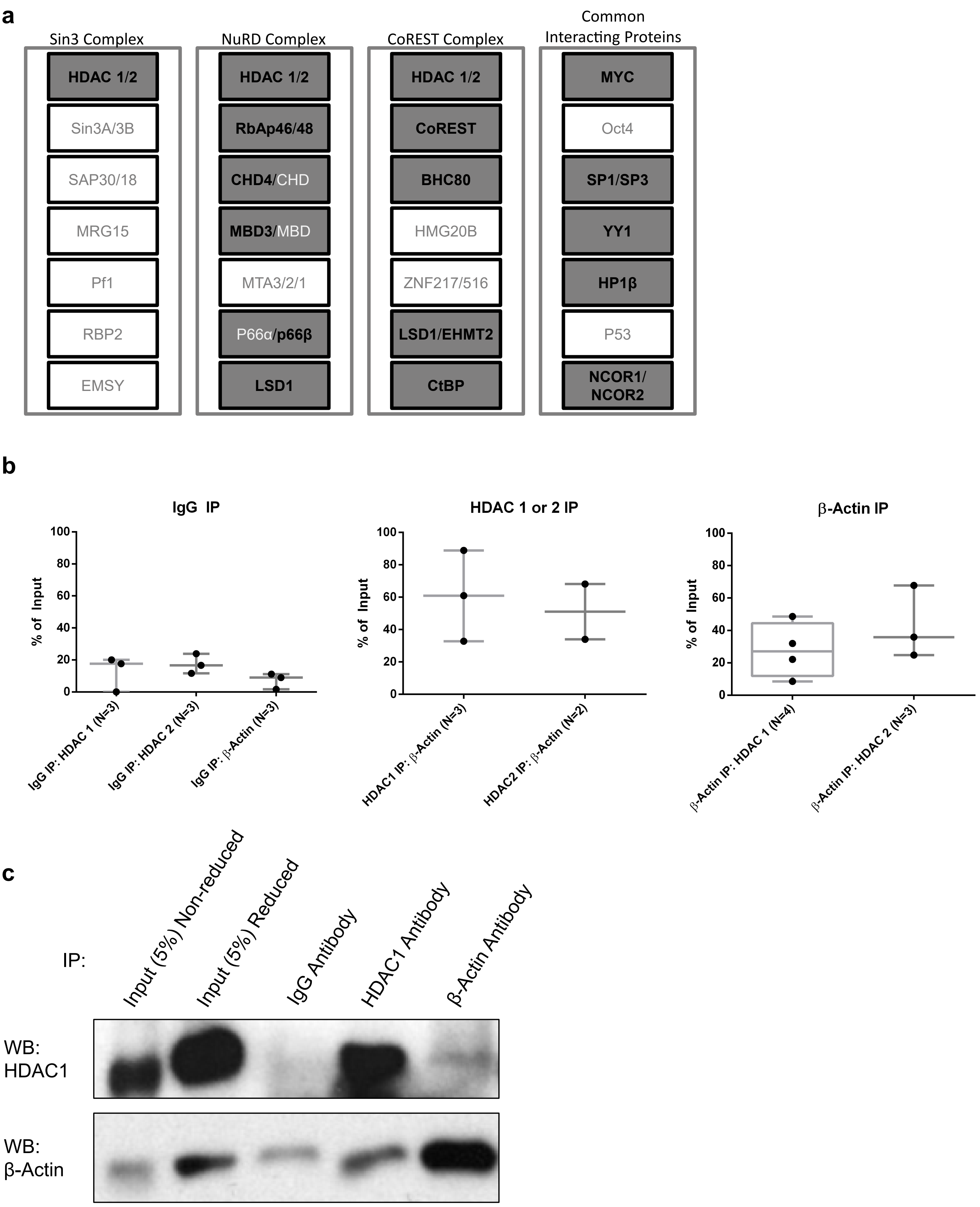

Supplementary Figure S2

a

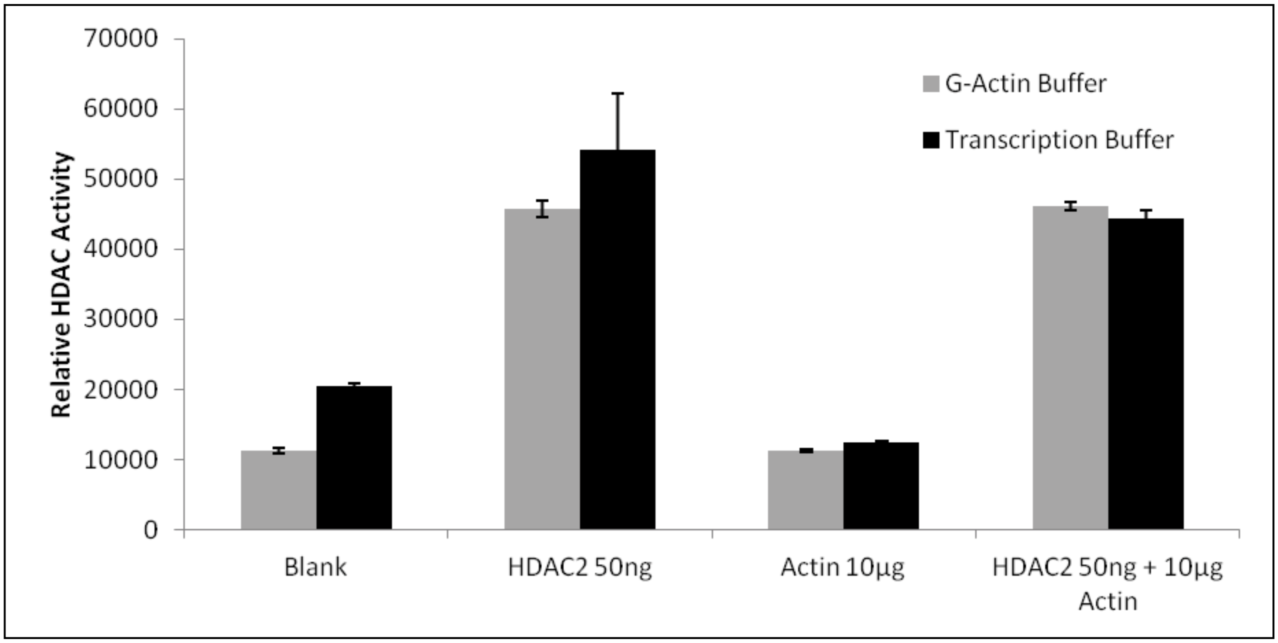

b

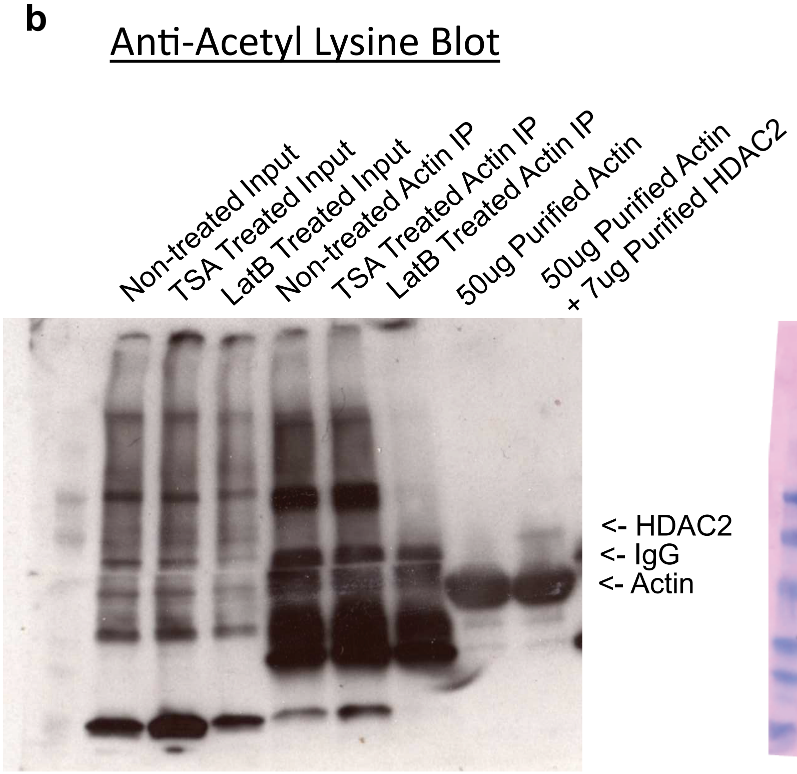

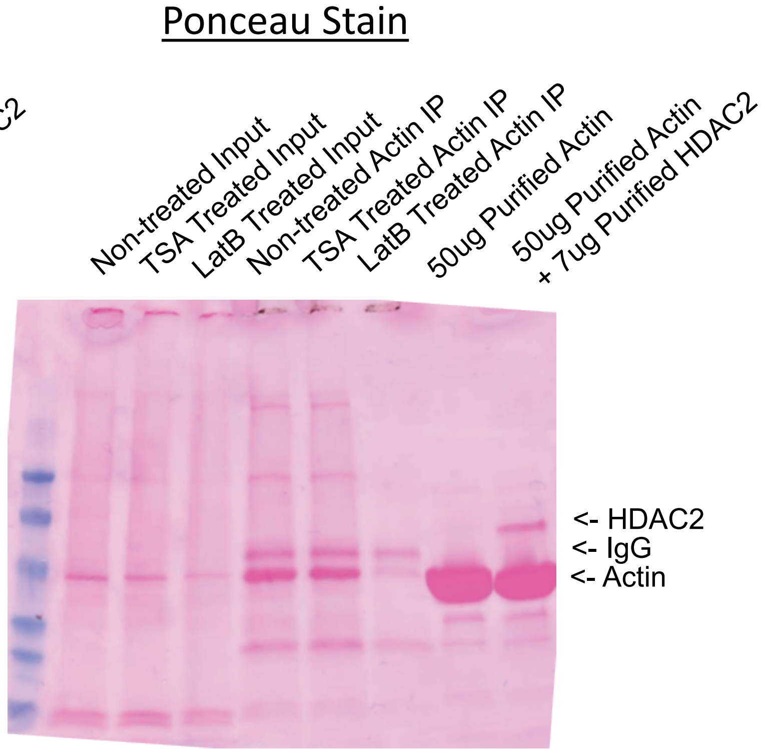

Supplementary Figure S3

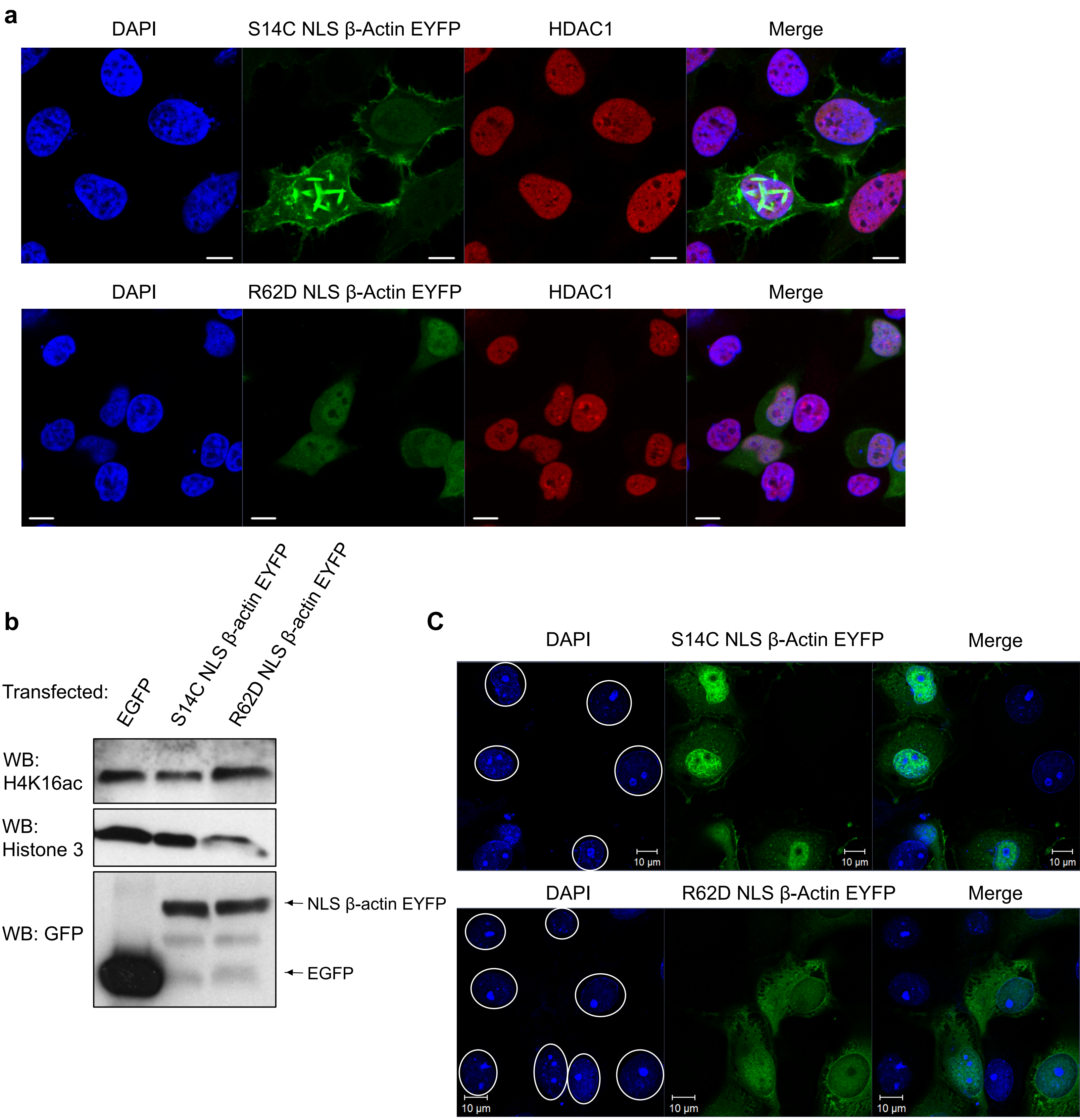

Identified Proteins (687) - Actin Pulldown #1

Proteins Within 95% Threshold	Accession Number	Molecular Weight	Assigned Function	Scaffold Protein Identification Probability	# Unique Identified Peptides	Percent Coverage of Protein	Percentage of Total Spectra	# Spectrum Counts
Elongation factor 1-alpha 1 OS=Homo sapiens GN=EEF1A1 PE=1 SV=1	EF1A1_HUMAN	50 kDa	Other	100%	14	58.40%	3.30%	702
~~Actin, cytoplasmic 1 OS=Homo sapiens GN=ACTB PE=1 SV=1~~	ACTB_HUMAN (+1)	42 kDa	actin	100%	11	55.50%	1.10%	241
~~Actin, alpha cardiac muscle 1 OS=Homo sapiens GN=ACTC1 PE=1 SV=1~~	ACTC_HUMAN	42 kDa	Actin	100%	3	33.40%	0.16%	34
~~Neuroblast differentiation-associated protein AHNAK OS=Homo sapiens GN=AHNAK PE=1 S~~	AHNK_HUMAN	629 kDa	Actin Binding Protein	100%	69	18.80%	1.30%	285
Plectin-1 OS=Homo sapiens GN=PLEC1 PE=1 SV=3	PLEC1_HUMAN	532 kDa	Actin Binding Protein	100%	53	19.10%	1.30%	278
Desmoplakin OS=Homo sapiens GN=DSP PE=1 SV=3	DESP_HUMAN	332 kDa	Actin Binding Protein	100%	21	14.80%	0.50%	107
Cytoplasmic dynein 1 heavy chain 1 OS=Homo sapiens GN=DYNC1H1 PE=1 SV=5	DYHC1_HUMAN	532 kDa	Actin Binding Protein	100%	19	7.64%	0.49%	103
Rho GTPase-activating protein 17 OS=Homo sapiens GN=ARHGAP17 PE=1 SV=1	RHG17_HUMAN	95 kDa	Actin Binding Protein	100%	5	11.80%	0.18%	38
Cytoplasmic dynein 1 light intermediate chain 1 OS=Homo sapiens GN=DYNC1LI1 PE=1 SV=	DC1L1_HUMAN	57 kDa	Actin Binding Protein	100%	5	18.00%	0.15%	31
Dynamin-2 OS=Homo sapiens GN=DNM2 PE=1 SV=2	DYN2_HUMAN	98 kDa	Actin Binding Protein	100%	8	13.90%	0.13%	27
Cytoplasmic dynein 1 intermediate chain 2 OS=Homo sapiens GN=DYNC1I2 PE=1 SV=3	DC1I2_HUMAN	71 kDa	Actin Binding Protein	100%	5	17.60%	0.13%	27
Dynein light chain 1, cytoplasmic OS=Homo sapiens GN=DYNLL1 PE=1 SV=1	DYL1_HUMAN	10 kDa	Actin Binding Protein	100%	2	42.70%	0.11%	24
LIM and calponin homology domains-containing protein 1 OS=Homo sapiens GN=LIMCH1 F	LIMC1_HUMAN	122 kDa	Actin Binding Protein	100%	2	5.72%	0.11%	23
Annexin A2 OS=Homo sapiens GN=ANXA2 PE=1 SV=2	ANXA2_HUMAN	39 kDa	Actin Binding Protein	100%	4	17.10%	0.10%	21
Catenin delta-1 OS=Homo sapiens GN=CTNND1 PE=1 SV=1	CTND1_HUMAN	108 kDa	Actin Binding Protein	100%	4	8.26%	0.09%	20
Myopalladin OS=Homo sapiens GN=MYPN PE=1 SV=2	MYPN_HUMAN	145 kDa	Actin Binding Protein	100%	3	5.08%	0.09%	20
Brain-specific angiogenesis inhibitor 1-associated protein 2-like protein 1 OS=Homo sapien	BI2L1_HUMAN	57 kDa	Actin Binding Protein	100%	2	8.81%	0.09%	18
Cytoplasmic dynein 1 light intermediate chain 2 OS=Homo sapiens GN=DYNC1LI2 PE=1 SV=	DC1L2_HUMAN	54 kDa	Actin Binding Protein	100%	3	16.10%	0.08%	17
PDZ and LIM domain protein 7 OS=Homo sapiens GN=PDLIM7 PE=1 SV=1	PDLI7_HUMAN	50 kDa	Actin Binding Protein	100%	5	24.50%	0.08%	16
Actin-like protein 6A OS=Homo sapiens GN=ACTL6A PE=1 SV=1	ACL6A_HUMAN	47 kDa	Actin Binding Protein	100%	4	18.60%	0.07%	15
Alpha-centractin OS=Homo sapiens GN=ACTR1A PE=1 SV=1	ACTZ_HUMAN	43 kDa	Actin Binding Protein	100%	3	18.10%	0.06%	12
Actin-binding protein anillin OS=Homo sapiens GN=ANLN PE=1 SV=2	ANLN_HUMAN	124 kDa	Actin Binding Protein	100%	3	5.43%	0.05%	11
Spectrin alpha chain, brain OS=Homo sapiens GN=SPTAN1 PE=1 SV=3	SPTA2_HUMAN	285 kDa	Actin Binding Protein	100%	2	1.70%	0.05%	10
Four and a half LIM domains protein 2 OS=Homo sapiens GN=FHL2 PE=1 SV=3	FHL2_HUMAN	32 kDa	Actin Binding Protein	100%	2	16.80%	0.04%	9
Serine/threonine-protein kinase OSR1 OS=Homo sapiens GN=OXSR1 PE=1 SV=1	OXSR1_HUMAN	58 kDa	Actin Binding Protein	100%	2	8.92%	0.02%	5
Ankyrin-3 OS=Homo sapiens GN=ANK3 PE=1 SV=1	ANK3_HUMAN	480 kDa	Actin Binding Protein	100%	2	0.73%	0.01%	3
Cordon-bleu protein-like 1 OS=Homo sapiens GN=COBLL1 PE=1 SV=2	COBL1_HUMAN	132 kDa	Actin Binding Protein	99%	2	5.32%	0.08%	17
Apoptosis inhibitor 5 OS=Homo sapiens GN=API5 PE=1 SV=2	API5_HUMAN	58 kDa	Cell Cycle/Apoptosis	100%	5	17.50%	0.14%	29
S-phase kinase-associated protein 1 OS=Homo sapiens GN=SKP1 PE=1 SV=2	SKP1_HUMAN	19 kDa	Cell Cycle/Apoptosis	100%	3	20.20%	0.13%	28
Serine/threonine-protein phosphatase 1 regulatory subunit 10 OS=Homo sapiens GN=PPP1	PP1RA_HUMAN	99 kDa	Cell Cycle/Apoptosis	100%	3	6.06%	0.07%	14
Death-inducer obliterator 1 OS=Homo sapiens GN=DIDO1 PE=1 SV=5	DIDO1_HUMAN	244 kDa	Cell Cycle/Apoptosis	100%	3	2.46%	0.06%	13
~~Heat shock cognate 71 kDa protein OS=Homo sapiens GN=HSPA8 PE=1 SV=1~~	HSP7C_HUMAN	71 kDa	Chaperone/Heatshock	100%	15	34.50%	0.59%	125
DnaJ homolog subfamily A member 1 OS=Homo sapiens GN=DNAJA1 PE=1 SV=2	DNJA1_HUMAN	45 kDa	Chaperone/Heatshock	100%	10	40.60%	0.47%	99
Heat shock 70 kDa protein 1 OS=Homo sapiens GN=HSPA1A PE=1 SV=5	HSP71_HUMAN	70 kDa	Chaperone/Heatshock	100%	14	38.20%	0.44%	94
T-complex protein 1 subunit delta OS=Homo sapiens GN=CCT4 PE=1 SV=4	TCPD_HUMAN	58 kDa	Chaperone/Heatshock	100%	10	37.50%	0.32%	68
DnaJ homolog subfamily A member 2 OS=Homo sapiens GN=DNAJA2 PE=1 SV=1	DNJA2_HUMAN	46 kDa	Chaperone/Heatshock	100%	7	35.70%	0.30%	63
~~78 kDa glucose-regulated protein OS=Homo sapiens GN=HSPA5 PE=1 SV=2~~	GRP78_HUMAN	72 kDa	Chaperone/Heatshock	100%	12	25.80%	0.29%	62
Heat shock protein beta-1 OS=Homo sapiens GN=HSPB1 PE=1 SV=2	HSPB1_HUMAN	23 kDa	Chaperone/Heatshock	100%	5	36.10%	0.24%	51
T-complex protein 1 subunit zeta OS=Homo sapiens GN=CCT6A PE=1 SV=3	TCPZ_HUMAN	58 kDa	Chaperone/Heatshock	100%	6	22.00%	0.21%	44
~~60 kDa heat shock protein, mitochondrial OS=Homo sapiens GN=HSPD1 PE=1 SV=2~~	CH60_HUMAN	61 kDa	Chaperone/Heatshock	100%	8	23.70%	0.17%	36
~~T-complex protein 1 subunit beta OS=Homo sapiens GN=CCT2 PE=1 SV=4~~	TCPB_HUMAN	57 kDa	Chaperone/Heatshock	100%	6	20.60%	0.15%	31
Prefoldin subunit 5 OS=Homo sapiens GN=PFDN5 PE=1 SV=2	PFD5_HUMAN	17 kDa	Chaperone/Heatshock	100%	2	24.00%	0.11%	23
Peptidyl-prolyl cis-trans isomerase A OS=Homo sapiens GN=PPIA PE=1 SV=2	PPIA_HUMAN	18 kDa	Chaperone/Heatshock	100%	4	41.20%	0.09%	18
DnaJ homolog subfamily A member 4 OS=Homo sapiens GN=DNAJA4 PE=1 SV=1	DNJA4_HUMAN	45 kDa	Chaperone/Heatshock	100%	3	14.40%	0.08%	17
Prefoldin subunit 3 OS=Homo sapiens GN=VBP1 PE=1 SV=3	PFD3_HUMAN	23 kDa	Chaperone/Heatshock	100%	3	21.30%	0.08%	16
T-complex protein 1 subunit gamma OS=Homo sapiens GN=CCT3 PE=1 SV=4	TCPG_HUMAN	61 kDa	Chaperone/Heatshock	100%	6	19.40%	0.07%	15
T-complex protein 1 subunit eta OS=Homo sapiens GN=CCT7 PE=1 SV=2	TCPH_HUMAN	59 kDa	Chaperone/Heatshock	100%	4	14.90%	0.07%	15
DnaJ homolog subfamily C member 9 OS=Homo sapiens GN=DNAJC9 PE=1 SV=1	DNJC9_HUMAN	30 kDa	Chaperone/Heatshock	100%	3	18.50%	0.07%	15
T-complex protein 1 subunit epsilon OS=Homo sapiens GN=CCT5 PE=1 SV=1	TCPE_HUMAN	60 kDa	Chaperone/Heatshock	100%	3	9.43%	0.05%	11
Heat shock protein HSP 90-beta OS=Homo sapiens GN=HSP90AB1 PE=1 SV=4	HS90B_HUMAN	83 kDa	Chaperone/Heatshock	100%	4	9.12%	0.05%	10
Prefoldin subunit 2 OS=Homo sapiens GN=PFDN2 PE=1 SV=1	PFD2_HUMAN	17 kDa	Chaperone/Heatshock	100%	3	29.20%	0.05%	10
BAG family molecular chaperone regulator 2 OS=Homo sapiens GN=BAG2 PE=1 SV=1	BAG2_HUMAN	24 kDa	Chaperone/Heatshock	100%	2	13.30%	0.02%	4
Protein KIAA1967 OS=Homo sapiens GN=KIAA1967 PE=1 SV=2	K1967_HUMAN	103 kDa	Chromatin Remodeler	100%	14	29.50%	0.46%	98
RuvB-like 1 OS=Homo sapiens GN=RUVBL1 PE=1 SV=1	RUVB1_HUMAN	50 kDa	Chromatin Remodeler	100%	13	51.10%	0.46%	98
Histone deacetylase 2 OS=Homo sapiens GN=HDAC2 PE=1 SV=2	HDAC2_HUMAN	55 kDa	Chromatin Remodeler	100%	9	33.80%	0.32%	68
Nuclear receptor corepressor 2 OS=Homo sapiens GN=NCOR2 PE=1 SV=1	NCOR2_HUMAN	274 kDa	Chromatin Remodeler	100%	13	10.20%	0.29%	61
Chromodomain-helicase-DNA-binding protein 8 OS=Homo sapiens GN=CHD8 PE=1 SV=3	CHD8_HUMAN	262 kDa	Chromatin Remodeler	100%	14	10.30%	0.28%	59
Lysine-specific histone demethylase 1 OS=Homo sapiens GN=KDM1 PE=1 SV=2	O60341|KDM1_HUMAN	?	Chromatin Remodeler	100%	13	0.00%	0.21%	44
Chromodomain-helicase-DNA-binding protein 4 OS=Homo sapiens GN=CHD4 PE=1 SV=1	CHD4_HUMAN	218 kDa	Chromatin Remodeler	100%	7	6.96%	0.20%	43
SWI/SNF-related matrix-associated actin-dependent regulator of chromatin subfamily E me	SMCE1_HUMAN	47 kDa	Chromatin Remodeler	100%	6	22.90%	0.16%	33
Histone-lysine N-methyltransferase SETD1A OS=Homo sapiens GN=SETD1A PE=1 SV=3	SET1A_HUMAN	186 kDa	Chromatin Remodeler	100%	6	7.62%	0.13%	28
Protein dpy-30 homolog OS=Homo sapiens GN=DPY30 PE=1 SV=1	DPY30_HUMAN	11 kDa	Chromatin Remodeler	100%	2	36.40%	0.12%	25
Nuclear receptor corepressor 1 OS=Homo sapiens GN=NCOR1 PE=1 SV=2	NCOR1_HUMAN	270 kDa	Chromatin Remodeler	100%	5	4.84%	0.11%	23
Retinoblastoma-binding protein 5 OS=Homo sapiens GN=RBBP5 PE=1 SV=2	RBBP5_HUMAN	59 kDa	Chromatin Remodeler	100%	5	17.10%	0.10%	21
Uncharacterized potential DNA-binding protein C17orf49 OS=Homo sapiens GN=C17orf49	CQ049_HUMAN	18 kDa	Chromatin Remodeler	100%	5	41.90%	0.09%	20
Lysine-specific demethylase 3B OS=Homo sapiens GN=KDM3B PE=1 SV=1	Q7LBC6|KDM3B_HUMAN	?	Chromatin Remodeler	100%	5	0.00%	0.09%	19
Histone deacetylase 1 OS=Homo sapiens GN=HDAC1 PE=1 SV=1	HDAC1_HUMAN	55 kDa	Chromatin Remodeler	100%	4	22.60%	0.08%	16
Probable JmjC domain-containing histone demethylation protein 2C OS=Homo sapiens GN:	JHD2C_HUMAN	285 kDa	Chromatin Remodeler	100%	2	1.54%	0.08%	16
Methyl-CpG-binding domain protein 3 OS=Homo sapiens GN=MBD3 PE=1 SV=1	MBD3_HUMAN	33 kDa	Chromatin Remodeler	100%	2	11.30%	0.08%	16
SWI/SNF complex subunit SMARCC1 OS=Homo sapiens GN=SMARCC1 PE=1 SV=3	SMRC1_HUMAN	123 kDa	Chromatin Remodeler	100%	4	6.88%	0.07%	15
Methylated-DNA--protein-cysteine methyltransferase OS=Homo sapiens GN=MGMT PE=1 !	MGMT_HUMAN	22 kDa	Chromatin Remodeler	100%	2	17.40%	0.07%	15
PHD finger protein 21A OS=Homo sapiens GN=PHF21A PE=1 SV=1	PF21A_HUMAN	75 kDa	Chromatin Remodeler	100%	3	10.10%	0.07%	14
Histone-binding protein RBBP4 OS=Homo sapiens GN=RBBP4 PE=1 SV=3	RBBP4_HUMAN	48 kDa	Chromatin Remodeler	100%	3	16.90%	0.06%	13
Protein SET OS=Homo sapiens GN=SET PE=1 SV=3	SET_HUMAN	33 kDa	Chromatin Remodeler	100%	2	11.00%	0.06%	13
Genetic suppressor element 1 OS=Homo sapiens GN=GSE1 PE=2 SV=3	GSE1_HUMAN	136 kDa	Chromatin Remodeler	100%	3	6.00%	0.06%	12
Set1/Ash2 histone methyltransferase complex subunit ASH2 OS=Homo sapiens GN=ASH2L	ASH2L_HUMAN	69 kDa	Chromatin Remodeler	100%	3	11.10%	0.05%	11
WD repeat-containing protein 5 OS=Homo sapiens GN=WDR5 PE=1 SV=1	WDR5_HUMAN	37 kDa	Chromatin Remodeler	100%	3	12.30%	0.05%	10
WW domain-containing adapter protein with coiled-coil OS=Homo sapiens GN=WAC PE=1	WAC_HUMAN	71 kDa	Chromatin Remodeler	100%	3	10.20%	0.05%	10
Protein Wiz OS=Homo sapiens GN=WIZ PE=1 SV=2	WIZ_HUMAN	179 kDa	Chromatin Remodeler	100%	2	2.67%	0.05%	10
MRG-binding protein OS=Homo sapiens GN=MRGBP PE=1 SV=1	MRGBP_HUMAN	22 kDa	Chromatin Remodeler	100%	2	16.70%	0.04%	8
BCL-6 corepressor OS=Homo sapiens GN=BCOR PE=1 SV=1	BCOR_HUMAN	192 kDa	Chromatin Remodeler	100%	3	4.56%	0.03%	7
Bromodomain-containing protein 8 OS=Homo sapiens GN=BRD8 PE=1 SV=1	BRD8_HUMAN	135 kDa	Chromatin Remodeler	100%	2	3.56%	0.03%	6
YEATS domain-containing protein 2 OS=Homo sapiens GN=YEATS2 PE=1 SV=2	YETS2_HUMAN	151 kDa	Chromatin Remodeler	100%	2	2.32%	0.02%	5
REST corepressor 1 OS=Homo sapiens GN=RCOR1 PE=1 SV=1	RCOR1_HUMAN	53 kDa	DNA Damage	100%	9	38.80%	0.33%	69
DNA damage-binding protein 1 OS=Homo sapiens GN=DDB1 PE=1 SV=1	DDB1_HUMAN	127 kDa	DNA Damage	100%	11	16.40%	0.24%	52
RuvB-like 2 OS=Homo sapiens GN=RUVBL2 PE=1 SV=3	RUVB2_HUMAN	51 kDa	DNA Damage	100%	8	23.30%	0.21%	45
DNA repair protein RAD50 OS=Homo sapiens GN=RAD50 PE=1 SV=1	RAD50_HUMAN	154 kDa	DNA Damage	100%	7	9.53%	0.16%	35
Double-strand break repair protein MRE11A OS=Homo sapiens GN=MRE11A PE=1 SV=3	MRE11_HUMAN	81 kDa	DNA Damage	100%	6	13.70%	0.15%	31
DNA-dependent protein kinase catalytic subunit OS=Homo sapiens GN=PRKDC PE=1 SV=3	PRKDC_HUMAN	469 kDa	DNA Damage	100%	5	1.82%	0.12%	26
Structural maintenance of chromosomes protein 4 OS=Homo sapiens GN=SMC4 PE=1 SV=2	SMC4_HUMAN	147 kDa	DNA Damage	100%	3	3.88%	0.12%	25
ATP-dependent DNA helicase 2 subunit 1 OS=Homo sapiens GN=XRCC6 PE=1 SV=2	KU70_HUMAN	70 kDa	DNA Damage	100%	3	8.54%	0.09%	20
ATP-dependent RNA helicase DDX39 OS=Homo sapiens GN=DDX39 PE=1 SV=2	DDX39_HUMAN	49 kDa	DNA Damage	100%	7	26.20%	0.09%	19
ATP-dependent RNA helicase DDX1 OS=Homo sapiens GN=DDX1 PE=1 SV=2	DDX1_HUMAN	82 kDa	DNA Damage	100%	4	9.05%	0.09%	19
Fanconi anemia group I protein OS=Homo sapiens GN=FANCI PE=1 SV=4	FANCI_HUMAN	149 kDa	DNA Damage	100%	3	4.07%	0.09%	18
Replication protein A 14 kDa subunit OS=Homo sapiens GN=RPA3 PE=1 SV=1	RFA3_HUMAN	14 kDa	DNA Damage	100%	2	33.10%	0.08%	16
DNA mismatch repair protein Mlh1 OS=Homo sapiens GN=MLH1 PE=1 SV=1	MLH1_HUMAN	85 kDa	DNA Damage	100%	3	7.54%	0.07%	15
ATP-dependent DNA helicase 2 subunit 2 OS=Homo sapiens GN=XRCC5 PE=1 SV=3	KU86_HUMAN	83 kDa	DNA Damage	100%	4	11.20%	0.07%	14
Integrator complex subunit 3 OS=Homo sapiens GN=INTS3 PE=1 SV=1	INT3_HUMAN	118 kDa	DNA Damage	100%	3	5.08%	0.05%	11
Replication factor C subunit 4 OS=Homo sapiens GN=RFC4 PE=1 SV=2	RFC4_HUMAN	40 kDa	DNA Replication Facto	100%	9	40.50%	0.25%	53
Replication factor C subunit 2 OS=Homo sapiens GN=RFC2 PE=1 SV=3	RFC2_HUMAN	39 kDa	DNA Replication Facto	100%	5	24.90%	0.17%	37
Nucleosome assembly protein 1-like 1 OS=Homo sapiens GN=NAP1L1 PE=1 SV=1	NP1L1_HUMAN	45 kDa	DNA Replication Facto	100%	2	7.42%	0.05%	10
Origin recognition complex subunit 2 OS=Homo sapiens GN=ORC2L PE=1 SV=2	ORC2_HUMAN	66 kDa	DNA Replication Facto	100%	2	5.20%	0.05%	10
Coatomer subunit gamma OS=Homo sapiens GN=COPG PE=1 SV=1	COPG_HUMAN	98 kDa	ER Transport	100%	11	24.60%	0.38%	81
Coatomer subunit beta OS=Homo sapiens GN=COPB1 PE=1 SV=3	COPB_HUMAN	107 kDa	ER Transport	100%	7	12.70%	0.12%	25
Coatomer subunit delta OS=Homo sapiens GN=ARCN1 PE=2 SV=1	COPD_HUMAN	57 kDa	ER Transport	100%	5	12.90%	0.12%	25
Coatomer subunit epsilon OS=Homo sapiens GN=COPE PE=2 SV=3	COPE_HUMAN	34 kDa	ER Transport	100%	5	31.20%	0.10%	21
Calumenin OS=Homo sapiens GN=CALU PE=1 SV=2	CALU_HUMAN	37 kDa	ER Transport	100%	2	14.60%	0.09%	18
Spastin OS=Homo sapiens GN=SPAST PE=1 SV=1	SPAST_HUMAN	67 kDa	ER Transport	100%	2	6.66%	0.09%	18
AP-1 complex subunit beta-1 OS=Homo sapiens GN=AP1B1 PE=2 SV=1	AP1B1_HUMAN	105 kDa	ER Transport	100%	2	4.95%	0.06%	12
Lamina-associated polypeptide 2 isoform alpha OS=Homo sapiens GN=TMPO PE=1 SV=2	LAP2A_HUMAN	75 kDa	Lamina Protein	100%	14	40.20%	0.87%	185
Lamin-A/C OS=Homo sapiens GN=LMNA PE=1 SV=1	LMNA_HUMAN	74 kDa	Lamina Protein	100%	6	19.30%	0.28%	59
Lamin-B1 OS=Homo sapiens GN=LMNB1 PE=1 SV=2	LMNB1_HUMAN	66 kDa	Lamina Protein	100%	5	16.60%	0.15%	32
Large proline-rich protein BAT3 OS=Homo sapiens GN=BAT3 PE=1 SV=2	BAT3_HUMAN	119 kDa	Mitotic Machinery	100%	11	19.70%	0.34%	72
Biorientation of chromosomes in cell division protein 1-like OS=Homo sapiens GN=BOD1L F	Q8NFC6|BOD1L_HUMAN	?	Mitotic Machinery	100%	4	0.00%	0.10%	22
Sister chromatid cohesion protein DCC1 OS=Homo sapiens GN=DSCC1 PE=1 SV=2	DCC1_HUMAN	45 kDa	Mitotic Machinery	100%	5	23.20%	0.09%	20
Nuclear mitotic apparatus protein 1 OS=Homo sapiens GN=NUMA1 PE=1 SV=2	NUMA1_HUMAN	238 kDa	Mitotic Machinery	100%	3	3.17%	0.05%	11
Structural maintenance of chromosomes protein 3 OS=Homo sapiens GN=SMC3 PE=1 SV=2	SMC3_HUMAN	142 kDa	Mitotic Machinery	100%	3	3.78%	0.05%	10
Kinetochore-associated protein DSN1 homolog OS=Homo sapiens GN=DSN1 PE=1 SV=2	DSN1_HUMAN	40 kDa	Mitotic Machinery	100%	2	8.99%	0.05%	10
Anaphase-promoting complex subunit 1 OS=Homo sapiens GN=ANAPC1 PE=1 SV=1	APC1_HUMAN	217 kDa	Mitotic Machinery	100%	3	2.78%	0.04%	8
Regulator of chromosome condensation OS=Homo sapiens GN=RCC1 PE=1 SV=1	RCC1_HUMAN	45 kDa	Mitotic Modulator	100%	2	8.08%	0.02%	5
Mitotic spindle assembly checkpoint protein MAD1 OS=Homo sapiens GN=MAD1L1 PE=1 S'	MD1L1_HUMAN	83 kDa	Mitotic Modulator	99%	2	6.13%	0.03%	7
Condensin-2 complex subunit H2 OS=Homo sapiens GN=NCAPH2 PE=1 SV=1	CNDH2_HUMAN	68 kDa	Mitotic Modulator	96%	2	8.60%	0.01%	2
14-3-3 protein beta/alpha OS=Homo sapiens GN=YWHAB PE=1 SV=3	1433B_HUMAN	28 kDa	Nuclear Transport	100%	5	27.60%	0.17%	37
Nucleophosmin OS=Homo sapiens GN=NPM1 PE=1 SV=2	NPM_HUMAN	33 kDa	Nuclear Transport	100%	3	18.40%	0.16%	33
14-3-3 protein gamma OS=Homo sapiens GN=YWHAG PE=1 SV=2	1433G_HUMAN	28 kDa	Nuclear Transport	100%	2	19.40%	0.14%	30
Ran GTPase-activating protein 1 OS=Homo sapiens GN=RANGAP1 PE=1 SV=1	RAGP1_HUMAN	64 kDa	Nuclear Transport	100%	6	15.00%	0.11%	23
Tumor protein D54 OS=Homo sapiens GN=TPD52L2 PE=1 SV=2	TPD54_HUMAN	22 kDa	Nuclear Transport	100%	5	35.90%	0.11%	23
14-3-3 protein epsilon OS=Homo sapiens GN=YWHAE PE=1 SV=1	1433E_HUMAN	29 kDa	Nuclear Transport	100%	3	22.70%	0.11%	23
14-3-3 protein zeta/delta OS=Homo sapiens GN=YWHAZ PE=1 SV=1	1433Z_HUMAN	28 kDa	Nuclear Transport	100%	2	16.70%	0.09%	18
Nuclear pore complex protein Nup107 OS=Homo sapiens GN=NUP107 PE=1 SV=1	NU107_HUMAN	106 kDa	Nuclear Transport	100%	4	8.86%	0.08%	16
Exportin-1 OS=Homo sapiens GN=XPO1 PE=1 SV=1	XPO1_HUMAN	123 kDa	Nuclear Transport	100%	3	5.88%	0.06%	13
Nuclear pore complex protein Nup155 OS=Homo sapiens GN=NUP155 PE=1 SV=1	NU155_HUMAN	155 kDa	Nuclear Transport	100%	2	3.52%	0.06%	12
Nuclear pore complex protein Nup133 OS=Homo sapiens GN=NUP133 PE=1 SV=2	NU133_HUMAN	129 kDa	Nuclear Transport	100%	2	3.98%	0.04%	9
Nuclear pore complex protein Nup88 OS=Homo sapiens GN=NUP88 PE=1 SV=2	NUP88_HUMAN	84 kDa	Nuclear Transport	100%	3	9.31%	0.04%	8
Nuclear pore complex protein Nup214 OS=Homo sapiens GN=NUP214 PE=1 SV=2	NU214_HUMAN	214 kDa	Nuclear Transport	100%	2	2.25%	0.04%	8
Protein SEC13 homolog OS=Homo sapiens GN=SEC13 PE=1 SV=3	SEC13_HUMAN	36 kDa	Nuclear Transport	100%	3	17.10%	0.03%	7
Importin subunit beta-1 OS=Homo sapiens GN=KPNB1 PE=1 SV=2	IMB1_HUMAN	97 kDa	Nuclear Transport	100%	3	5.37%	0.03%	7
Importin subunit alpha-3 OS=Homo sapiens GN=KPNA3 PE=1 SV=2	IMA3_HUMAN	58 kDa	Nuclear Transport	100%	2	10.40%	0.01%	3
~~Tubulin alpha-1B chain OS=Homo sapiens GN=TUBA1B PE=1 SV=1~~	TBA1B_HUMAN	50 kDa	Other	100%	13	47.20%	0.68%	145
Translational activator GCN1 OS=Homo sapiens GN=GCN1L1 PE=1 SV=5	GCN1L_HUMAN	293 kDa	Other	100%	19	12.50%	0.57%	122
~~Tubulin beta chain OS=Homo sapiens GN=TUBB PE=1 SV=2~~	TBB5_HUMAN	50 kDa	Other	100%	15	55.20%	0.56%	118
Keratin, type I cytoskeletal 10 OS=Homo sapiens GN=KRT10 PE=1 SV=4	K1C10_HUMAN	60 kDa	Other	100%	8	22.90%	0.42%	90
Keratin, type I cytoskeletal 9 OS=Homo sapiens GN=KRT9 PE=1 SV=2	K1C9_HUMAN	62 kDa	Other	100%	8	28.10%	0.26%	55
Keratin, type II cytoskeletal 1 OS=Homo sapiens GN=KRT1 PE=1 SV=5	K2C1_HUMAN	66 kDa	Other	100%	5	12.00%	0.18%	38
Tubulin beta-4 chain OS=Homo sapiens GN=TUBB4 PE=1 SV=2	TBB4_HUMAN	50 kDa	Other	100%	5	55.20%	0.18%	38
Perilipin-3 OS=Homo sapiens GN=PLIN3 PE=1 SV=2	O60664|PLIN3_HUMAN	?	Other	100%	6	0.00%	0.16%	34
Probable UPF0334 kinase-like protein C1orf57 OS=Homo sapiens GN=C1orf57 PE=1 SV=1	CA057_HUMAN	21 kDa	Other	100%	5	42.10%	0.14%	30
Synaptic vesicle membrane protein VAT-1 homolog OS=Homo sapiens GN=VAT1 PE=1 SV=2	VAT1_HUMAN	42 kDa	Other	100%	6	29.50%	0.14%	29
Leucine-rich repeat-containing protein 40 OS=Homo sapiens GN=LRRC40 PE=1 SV=1	LRC40_HUMAN	68 kDa	Other	100%	9	24.30%	0.10%	22
Endophilin-A2 OS=Homo sapiens GN=SH3GL1 PE=1 SV=1	SH3G1_HUMAN	41 kDa	Other	100%	5	25.80%	0.10%	22
ADP-ribosylation factor 4 OS=Homo sapiens GN=ARF4 PE=1 SV=3	ARF4_HUMAN	21 kDa	Other	100%	3	25.60%	0.09%	18
~~Tubulin alpha-1C chain OS=Homo sapiens GN=TUBA1C PE=1 SV=1~~	TBA1C_HUMAN	50 kDa	Other	100%	3	47.40%	0.09%	18
SAM domain and HD domain-containing protein 1 OS=Homo sapiens GN=SAMHD1 PE=1 SV	SAMH1_HUMAN	72 kDa	Other	100%	3	8.15%	0.08%	17
ATP-binding cassette sub-family F member 3 OS=Homo sapiens GN=ABCF3 PE=2 SV=2	ABCF3_HUMAN	80 kDa	Other	100%	4	11.70%	0.07%	15
Clusterin OS=Homo sapiens GN=CLU PE=1 SV=1	CLUS_HUMAN	52 kDa	Other	100%	3	11.60%	0.07%	15
~~Nucleolin OS=Homo sapiens GN=NCL PE=1 SV=3~~	NUCL_HUMAN	77 kDa	Other	100%	2	5.07%	0.07%	15
Tetratricopeptide repeat protein 1 OS=Homo sapiens GN=TTC1 PE=1 SV=1	TTC1_HUMAN	34 kDa	Other	100%	2	18.80%	0.07%	15
Serum albumin OS=Homo sapiens GN=ALB PE=1 SV=2	ALBU_HUMAN	69 kDa	Other	100%	2	2.46%	0.07%	14
Eukaryotic initiation factor 4A-III OS=Homo sapiens GN=EIF4A3 PE=1 SV=4	IF4A3_HUMAN	47 kDa	Other	100%	3	11.90%	0.06%	13
Transferrin receptor protein 1 OS=Homo sapiens GN=TFRC PE=1 SV=2	TFR1_HUMAN	85 kDa	Other	100%	4	6.71%	0.06%	12
Keratin, type II cytoskeletal 2 epidermal OS=Homo sapiens GN=KRT2 PE=1 SV=1	K22E_HUMAN	66 kDa	Other	100%	2	6.05%	0.06%	12
60S acidic ribosomal protein P2 OS=Homo sapiens GN=RPLP2 PE=1 SV=1	RLA2_HUMAN	12 kDa	Other	100%	2	39.10%	0.05%	11
Spermatogenesis-associated protein 5 OS=Homo sapiens GN=SPATA5 PE=2 SV=2	SPAT5_HUMAN	98 kDa	Other	100%	2	4.14%	0.05%	11
Tubulin alpha-4A chain OS=Homo sapiens GN=TUBA4A PE=1 SV=1	TBA4A_HUMAN	50 kDa	Other	100%	2	45.50%	0.05%	11
Tubulin beta-6 chain OS=Homo sapiens GN=TUBB6 PE=1 SV=1	TBB6_HUMAN	50 kDa	Other	100%	2	24.70%	0.05%	11
Pinin OS=Homo sapiens GN=PNN PE=1 SV=4	PININ_HUMAN	82 kDa	Other	100%	2	6.28%	0.05%	10
40S ribosomal protein S7 OS=Homo sapiens GN=RPS7 PE=1 SV=1	RS7_HUMAN	22 kDa	Other	100%	2	26.80%	0.05%	10
Mitochondrial import inner membrane translocase subunit Tim13 OS=Homo sapiens GN=T	TIM13_HUMAN	11 kDa	Other	100%	2	26.30%	0.05%	10
Tubulin beta-3 chain OS=Homo sapiens GN=TUBB3 PE=1 SV=2	TBB3_HUMAN	50 kDa	Other	100%	3	32.90%	0.04%	9
UPF0582 protein C13orf37 OS=Homo sapiens GN=C13orf37 PE=2 SV=2	CM037_HUMAN	8 kDa	Other	100%	2	39.00%	0.04%	9
LanC-like protein 1 OS=Homo sapiens GN=LANCL1 PE=1 SV=1	LANC1_HUMAN	45 kDa	Other	100%	2	9.52%	0.04%	9
Reticulocalbin-1 OS=Homo sapiens GN=RCN1 PE=1 SV=1	RCN1_HUMAN	39 kDa	Other	100%	2	12.70%	0.04%	9
Leukocyte receptor cluster member 9 OS=Homo sapiens GN=LENG9 PE=2 SV=1	LENG9_HUMAN	51 kDa	Other	100%	2	9.81%	0.04%	9
Uncharacterized protein C10orf78 OS=Homo sapiens GN=C10orf78 PE=2 SV=1	CJ078_HUMAN	36 kDa	Other	100%	2	11.40%	0.04%	8
Protein FAM98B OS=Homo sapiens GN=FAM98B PE=1 SV=1	FA98B_HUMAN	37 kDa	Other	100%	2	13.00%	0.04%	8
Eukaryotic translation initiation factor 5A-1 OS=Homo sapiens GN=EIF5A PE=1 SV=2	IF5A1_HUMAN	17 kDa	Other	100%	2	26.00%	0.04%	8
tRNA (cytosine-5-)-methyltransferase NSUN2 OS=Homo sapiens GN=NSUN2 PE=1 SV=2	NSUN2_HUMAN	86 kDa	Other	100%	2	7.17%	0.04%	8
Transketolase OS=Homo sapiens GN=TKT PE=1 SV=3	TKT_HUMAN	68 kDa	Other	100%	2	6.90%	0.04%	8
60S ribosomal protein L23 OS=Homo sapiens GN=RPL23 PE=1 SV=1	RL23_HUMAN	15 kDa	Other	100%	2	25.00%	0.03%	7
Eukaryotic peptide chain release factor subunit 1 OS=Homo sapiens GN=ETF1 PE=1 SV=3	ERF1_HUMAN	49 kDa	Other	100%	2	11.40%	0.03%	6
EF-hand domain-containing protein D2 OS=Homo sapiens GN=EFHD2 PE=1 SV=1	EFHD2_HUMAN	27 kDa	Other	100%	3	14.20%	0.02%	5
Protein FAM128B OS=Homo sapiens GN=FAM128B PE=2 SV=1	F128B_HUMAN	16 kDa	Other	100%	2	24.70%	0.02%	5
Guanine nucleotide-binding protein G(I)/G(S)/G(T) subunit beta-1 OS=Homo sapiens GN=G	GBB1_HUMAN	37 kDa	Other	100%	2	12.10%	0.02%	5
SHC-transforming protein 1 OS=Homo sapiens GN=SHC1 PE=1 SV=4	SHC1_HUMAN	63 kDa	Other	100%	2	4.80%	0.02%	5
ADP-ribosylation factor GTPase-activating protein 2 OS=Homo sapiens GN=ARFGAP2 PE=1	ARFG2_HUMAN	57 kDa	Other	100%	3	10.90%	0.02%	4
Ras-related protein Rab-11A OS=Homo sapiens GN=RAB11A PE=1 SV=3	RB11A_HUMAN	24 kDa	Other	99%	2	18.10%	0.04%	9
FLYWCH family member 2 OS=Homo sapiens GN=FLYWCH2 PE=1 SV=1	FWCH2_HUMAN	15 kDa	Other	98%	2	32.10%	0.06%	13
E3 ubiquitin-protein ligase UBR5 OS=Homo sapiens GN=UBR5 PE=1 SV=2	UBR5_HUMAN	309 kDa	Protein Degredation	100%	16	10.70%	0.31%	66
F-box-like/WD repeat-containing protein TBL1XR1 OS=Homo sapiens GN=TBL1XR1 PE=1 SV	TBL1R_HUMAN	56 kDa	Protein Degredation	100%	7	25.70%	0.20%	42
E3 SUMO-protein ligase RanBP2 OS=Homo sapiens GN=RANBP2 PE=1 SV=2	RBP2_HUMAN	358 kDa	Protein Degredation	100%	11	6.11%	0.19%	41
E3 ubiquitin-protein ligase RING1 OS=Homo sapiens GN=RING1 PE=1 SV=2	RING1_HUMAN	42 kDa	Protein Degredation	100%	4	18.70%	0.11%	23
Nuclear-interacting partner of ALK OS=Homo sapiens GN=ZC3HC1 PE=1 SV=1	NIPA_HUMAN	55 kDa	Protein Degredation	100%	4	13.10%	0.09%	18
Ubiquitin carboxyl-terminal hydrolase 7 OS=Homo sapiens GN=USP7 PE=1 SV=2	UBP7_HUMAN	128 kDa	Protein Degredation	100%	4	7.53%	0.07%	14
Ubiquitin-associated protein 2-like OS=Homo sapiens GN=UBAP2L PE=1 SV=2	UBP2L_HUMAN	115 kDa	Protein Degredation	100%	4	6.16%	0.06%	13
F-box-like/WD repeat-containing protein TBL1X OS=Homo sapiens GN=TBL1X PE=1 SV=2	TBL1X_HUMAN	57 kDa	Protein Degredation	100%	3	21.70%	0.06%	13
E3 ubiquitin-protein ligase BRE1B OS=Homo sapiens GN=RNF40 PE=1 SV=3	BRE1B_HUMAN	114 kDa	Protein Degredation	100%	2	5.59%	0.04%	8
Ubiquitin-like protein 4A OS=Homo sapiens GN=UBL4A PE=1 SV=1	UBL4A_HUMAN	18 kDa	Protein Degredation	100%	2	19.70%	0.04%	8
Ubiquitin carboxyl-terminal hydrolase BAP1 OS=Homo sapiens GN=BAP1 PE=1 SV=2	BAP1_HUMAN	80 kDa	Protein Degredation	100%	2	5.62%	0.03%	7
Tripartite motif-containing protein 25 OS=Homo sapiens GN=TRIM25 PE=1 SV=1	TRI25_HUMAN	71 kDa	Protein Degredation	100%	2	4.44%	0.03%	7
Ubiquitin-conjugating enzyme E2 S OS=Homo sapiens GN=UBE2S PE=1 SV=2	UBE2S_HUMAN	24 kDa	Protein Degredation	100%	2	13.50%	0.03%	7
Proteasome activator complex subunit 2 OS=Homo sapiens GN=PSME2 PE=1 SV=3	PSME2_HUMAN	27 kDa	Protein Degredation	100%	2	14.60%	0.03%	6
Sequestosome-1 OS=Homo sapiens GN=SQSTM1 PE=1 SV=1	SQSTM_HUMAN	48 kDa	Protein Degredation	100%	2	11.60%	0.02%	4
26S proteasome non-ATPase regulatory subunit 2 OS=Homo sapiens GN=PSMD2 PE=1 SV=:	PSMD2_HUMAN	100 kDa	Protein Degredation	99%	2	4.07%	0.02%	4
Poly(rC)-binding protein 1 OS=Homo sapiens GN=PCBP1 PE=1 SV=2	PCBP1_HUMAN	37 kDa	RNA Binding Protein	100%	10	53.90%	0.55%	116
Heterogeneous nuclear ribonucleoprotein K OS=Homo sapiens GN=HNRNPK PE=1 SV=1	HNRPK_HUMAN	51 kDa	RNA Binding Protein	100%	6	18.60%	0.26%	56
Ribonucleoprotein PTB-binding 1 OS=Homo sapiens GN=RAVER1 PE=1 SV=1	RAVR1_HUMAN	64 kDa	RNA Binding Protein	100%	7	25.40%	0.26%	55
ATP-dependent RNA helicase DDX19A OS=Homo sapiens GN=DDX19A PE=1 SV=1	DD19A_HUMAN	54 kDa	RNA Binding Protein	100%	9	33.30%	0.24%	51
~~Poly(rC)-binding protein 2 OS=Homo sapiens GN=PCBP2 PE=1 SV=1~~	PCBP2_HUMAN	39 kDa	RNA Binding Protein	100%	6	36.70%	0.23%	49
Probable ATP-dependent RNA helicase DDX46 OS=Homo sapiens GN=DDX46 PE=1 SV=2	DDX46_HUMAN	117 kDa	RNA Binding Protein	100%	12	18.30%	0.23%	48
Heterogeneous nuclear ribonucleoprotein Q OS=Homo sapiens GN=SYNCRIP PE=1 SV=2	HNRPQ_HUMAN	70 kDa	RNA Binding Protein	100%	9	22.50%	0.21%	44
Heterogeneous nuclear ribonucleoprotein U OS=Homo sapiens GN=HNRNPU PE=1 SV=5	HNRPU_HUMAN	91 kDa	RNA Binding Protein	100%	7	15.70%	0.19%	40
Polypyrimidine tract-binding protein 1 OS=Homo sapiens GN=PTBP1 PE=1 SV=1	PTBP1_HUMAN	57 kDa	RNA Binding Protein	100%	7	24.70%	0.18%	39
~~Heterogeneous nuclear ribonucleoprotein H OS=Homo sapiens GN=HNRNPH1 PE=1 SV=4~~	HNRH1_HUMAN	49 kDa	RNA Binding Protein	100%	7	29.20%	0.17%	37
Putative heterogeneous nuclear ribonucleoprotein A1-like protein 3 OS=Homo sapiens GN:	RA1L3_HUMAN (+2)	34 kDa	RNA Binding Protein	100%	4	17.80%	0.15%	32
Cleavage stimulation factor 64 kDa subunit OS=Homo sapiens GN=CSTF2 PE=1 SV=1	CSTF2_HUMAN	61 kDa	RNA Binding Protein	100%	7	26.20%	0.14%	29
Heterogeneous nuclear ribonucleoprotein L OS=Homo sapiens GN=HNRNPL PE=1 SV=1	HNRPL_HUMAN	60 kDa	RNA Binding Protein	100%	4	17.00%	0.13%	28
Cleavage stimulation factor 77 kDa subunit OS=Homo sapiens GN=CSTF3 PE=1 SV=1	CSTF3_HUMAN	83 kDa	RNA Binding Protein	100%	7	15.30%	0.12%	26
ATP-dependent RNA helicase DDX3X OS=Homo sapiens GN=DDX3X PE=1 SV=3	DDX3X_HUMAN	73 kDa	RNA Binding Protein	100%	6	12.10%	0.12%	25
~~Heterogeneous nuclear ribonucleoprotein F OS=Homo sapiens GN=HNRNPF PE=1 SV=3~~	HNRPF_HUMAN	46 kDa	RNA Binding Protein	100%	4	21.40%	0.12%	25
RNA-binding protein 39 OS=Homo sapiens GN=RBM39 PE=1 SV=2	RBM39_HUMAN	59 kDa	RNA Binding Protein	100%	5	15.70%	0.09%	20
~~Heterogeneous nuclear ribonucleoprotein D0 OS=Homo sapiens GN=HNRNPD PE=1 SV=1~~	HNRPD_HUMAN	38 kDa	RNA Binding Protein	100%	4	14.90%	0.09%	19
Heterogeneous nuclear ribonucleoprotein R OS=Homo sapiens GN=HNRNPR PE=1 SV=1	HNRPR_HUMAN	71 kDa	RNA Binding Protein	100%	5	14.80%	0.09%	18
Heterogeneous nuclear ribonucleoproteins A2/B1 OS=Homo sapiens GN=HNRNPA2B1 PE=:	ROA2_HUMAN	37 kDa	RNA Binding Protein	100%	3	11.60%	0.08%	16
Cleavage and polyadenylation specificity factor subunit 2 OS=Homo sapiens GN=CPSF2 PE=	CPSF2_HUMAN	88 kDa	RNA Binding Protein	100%	3	6.01%	0.05%	11
Zinc finger CCCH domain-containing protein 11A OS=Homo sapiens GN=ZC3H11A PE=1 SV=	ZC11A_HUMAN	89 kDa	RNA Binding Protein	100%	3	6.42%	0.05%	10
KH domain-containing, RNA-binding, signal transduction-associated protein 1 OS=Homo sa	KHDR1_HUMAN	48 kDa	RNA Binding Protein	100%	2	7.67%	0.05%	10
Heterogeneous nuclear ribonucleoprotein M OS=Homo sapiens GN=HNRNPM PE=1 SV=3	HNRPM_HUMAN	78 kDa	RNA Binding Protein	100%	2	3.70%	0.03%	6
Polyamine-modulated factor 1 OS=Homo sapiens GN=PMF1 PE=1 SV=2	PMF1_HUMAN	23 kDa	RNA Binding Protein	100%	2	16.10%	0.02%	5
Cleavage and polyadenylation specificity factor subunit 6 OS=Homo sapiens GN=CPSF6 PE=	CPSF6_HUMAN	59 kDa	RNA Binding Protein	98%	2	7.44%	0.08%	16
Cleavage stimulation factor 50 kDa subunit OS=Homo sapiens GN=CSTF1 PE=1 SV=1	CSTF1_HUMAN	48 kDa	RNA Binding Protein	98%	2	9.05%	0.02%	5
Protein quaking OS=Homo sapiens GN=QKI PE=1 SV=1	QKI_HUMAN	38 kDa	RNA Binding Protein	97%	2	7.92%	0.02%	4
U5 small nuclear ribonucleoprotein 200 kDa helicase OS=Homo sapiens GN=ASCC3L1 PE=1	U520_HUMAN	245 kDa	Splicing Factor	100%	6	5.06%	0.16%	33
116 kDa U5 small nuclear ribonucleoprotein component OS=Homo sapiens GN=EFTUD2 PE	U5S1_HUMAN	109 kDa	Splicing Factor	100%	5	9.05%	0.15%	31
Splicing factor, proline- and glutamine-rich OS=Homo sapiens GN=SFPQ PE=1 SV=2	SFPQ_HUMAN	76 kDa	Splicing Factor	100%	4	8.49%	0.12%	25
Pre-mRNA-splicing factor RBM22 OS=Homo sapiens GN=RBM22 PE=1 SV=1	RBM22_HUMAN	47 kDa	Splicing Factor	100%	3	14.80%	0.12%	25
Non-POU domain-containing octamer-binding protein OS=Homo sapiens GN=NONO PE=1 S	NONO_HUMAN	54 kDa	Splicing Factor	100%	3	12.50%	0.09%	19
Pre-mRNA-processing factor 19 OS=Homo sapiens GN=PRPF19 PE=1 SV=1	PRP19_HUMAN	55 kDa	Splicing Factor	100%	5	21.00%	0.09%	18
Splicing factor 3B subunit 2 OS=Homo sapiens GN=SF3B2 PE=1 SV=1	SF3B2_HUMAN	98 kDa	Splicing Factor	100%	4	8.94%	0.08%	17
Nuclease-sensitive element-binding protein 1 OS=Homo sapiens GN=YBX1 PE=1 SV=3	YBOX1_HUMAN	36 kDa	Splicing Factor	100%	2	14.50%	0.08%	17
Regulation of nuclear pre-mRNA domain-containing protein 2 OS=Homo sapiens GN=RPRD	RPRD2_HUMAN	156 kDa	Splicing Factor	100%	5	7.53%	0.06%	13
Muscleblind-like protein 1 OS=Homo sapiens GN=MBNL1 PE=1 SV=2	MBNL1_HUMAN	42 kDa	Splicing Factor	100%	4	20.90%	0.06%	13
Splicing factor 3A subunit 3 OS=Homo sapiens GN=SF3A3 PE=1 SV=1	SF3A3_HUMAN	59 kDa	Splicing Factor	100%	2	5.39%	0.06%	12
Splicing factor 3 subunit 1 OS=Homo sapiens GN=SF3A1 PE=1 SV=1	SF3A1_HUMAN	89 kDa	Splicing Factor	100%	3	6.81%	0.05%	10
Splicing factor 3B subunit 4 OS=Homo sapiens GN=SF3B4 PE=1 SV=1	SF3B4_HUMAN	44 kDa	Splicing Factor	100%	2	11.10%	0.05%	10
Splicing factor 3A subunit 2 OS=Homo sapiens GN=SF3A2 PE=1 SV=2	SF3A2_HUMAN	49 kDa	Splicing Factor	100%	2	7.54%	0.04%	9

Function	# of proteins
Actin Regulators	23
Cell Cycle/Apoptosis Proteins	4
Chaperone/Heat Shock Proteins	17
Chromatin Remodelers	31
DNA Damage Proteins	15
DNA Replication Factors	4
ER Transport Proteins	7
Lamin Proteins	3
Mitosis Associated Proteins	10
Nucleur Transport	16
Protein Degredation	16
mRNA Binding Proteins	24
Splicing Factors	18
Transcription Factors	49
Core Transcription Machinery	15
Unknown/Other	46

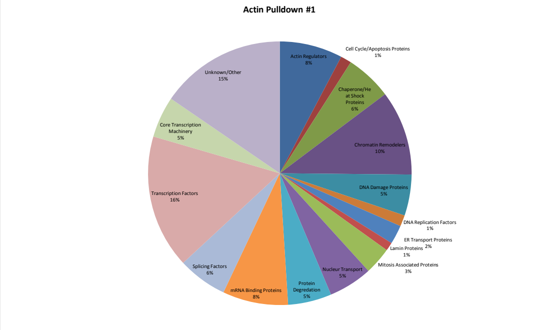

~~Poly(U)-binding-splicing factor PUF60 OS=Homo sapiens GN=PUF60 PE=1 SV=1~~	PUF60_HUMAN	60 kDa	Splicing Factor	100%	2	8.41%	0.04%	8
Serine/arginine repetitive matrix protein 2 OS=Homo sapiens GN=SRRM2 PE=1 SV=2	SRRM2_HUMAN	300 kDa	Splicing Factor	100%	3	1.67%	0.03%	7
Splicing factor U2AF 65 kDa subunit OS=Homo sapiens GN=U2AF2 PE=1 SV=4	U2AF2_HUMAN	54 kDa	Splicing Factor	100%	2	8.42%	0.03%	7
Splicing factor 1 OS=Homo sapiens GN=SF1 PE=1 SV=4	SF01_HUMAN	68 kDa	Splicing Factor	100%	2	7.36%	0.03%	6
U1 small nuclear ribonucleoprotein C OS=Homo sapiens GN=SNRPC PE=1 SV=1	RU1C_HUMAN	17 kDa	Splicing Factor	99%	2	18.90%	0.01%	2
Host cell factor OS=Homo sapiens GN=HCFC1 PE=1 SV=2	HCFC1_HUMAN	209 kDa	Transcription Factor	100%	26	26.60%	0.87%	185
Matrin-3 OS=Homo sapiens GN=MATR3 PE=1 SV=2	MATR3_HUMAN	95 kDa	Transcription Factor	100%	11	22.00%	0.61%	129
Transcription intermediary factor 1-beta OS=Homo sapiens GN=TRIM28 PE=1 SV=5	TIF1B_HUMAN	89 kDa	Transcription Factor	100%	16	45.60%	0.44%	93
Zinc finger protein 828 OS=Homo sapiens GN=ZNF828 PE=1 SV=2	ZN828_HUMAN	89 kDa	Transcription Factor	100%	12	30.00%	0.34%	73
RAS-responsive element-binding protein 1 OS=Homo sapiens GN=RREB1 PE=1 SV=3	RREB1_HUMAN	181 kDa	Transcription Factor	100%	9	13.20%	0.27%	58
Transcriptional repressor protein YY1 OS=Homo sapiens GN=YY1 PE=1 SV=2	TYY1_HUMAN	45 kDa	Transcription Factor	100%	10	33.60%	0.24%	52
Transcription factor Sp1 OS=Homo sapiens GN=SP1 PE=1 SV=3	SP1_HUMAN	81 kDa	Transcription Factor	100%	5	25.50%	0.21%	45
Zinc finger protein 24 OS=Homo sapiens GN=ZNF24 PE=1 SV=4	ZNF24_HUMAN	42 kDa	Transcription Factor	100%	5	28.50%	0.18%	39
Cyclic AMP-dependent transcription factor ATF-7 OS=Homo sapiens GN=ATF7 PE=1 SV=2	ATF7_HUMAN	53 kDa	Transcription Factor	100%	5	18.00%	0.18%	38
Pre-B-cell leukemia transcription factor 2 OS=Homo sapiens GN=PBX2 PE=1 SV=2	PBX2_HUMAN	46 kDa	Transcription Factor	100%	5	24.90%	0.11%	23
Homeobox protein Meis1 OS=Homo sapiens GN=MEIS1 PE=1 SV=1	MEIS1_HUMAN	43 kDa	Transcription Factor	100%	4	19.20%	0.10%	22
Pre-B-cell leukemia transcription factor 1 OS=Homo sapiens GN=PBX1 PE=1 SV=1	PBX1_HUMAN	47 kDa	Transcription Factor	100%	3	18.40%	0.10%	22
Zinc finger protein 143 OS=Homo sapiens GN=ZNF143 PE=2 SV=1	ZN143_HUMAN	68 kDa	Transcription Factor	100%	3	11.00%	0.10%	22
Alpha-taxilin OS=Homo sapiens GN=TXLNA PE=1 SV=3	TXLNA_HUMAN	62 kDa	Transcription Factor	100%	2	11.70%	0.10%	22
Interleukin enhancer-binding factor 2 OS=Homo sapiens GN=ILF2 PE=1 SV=2	ILF2_HUMAN	43 kDa	Transcription Factor	100%	4	22.10%	0.10%	21
Forkhead box protein P4 OS=Homo sapiens GN=FOXP4 PE=1 SV=1	FOXP4_HUMAN	73 kDa	Transcription Factor	100%	3	11.00%	0.09%	20
Dr1-associated corepressor OS=Homo sapiens GN=DRAP1 PE=1 SV=3	NC2A_HUMAN	22 kDa	Transcription Factor	100%	3	22.40%	0.09%	19
Protein Dr1 OS=Homo sapiens GN=DR1 PE=1 SV=1	NC2B_HUMAN	19 kDa	Transcription Factor	100%	3	34.70%	0.09%	18
Transcription factor AP-4 OS=Homo sapiens GN=TFAP4 PE=1 SV=2	TFAP4_HUMAN	39 kDa	Transcription Factor	100%	2	10.90%	0.09%	18
UPF0568 protein C14orf166 OS=Homo sapiens GN=C14orf166 PE=1 SV=1	CN166_HUMAN	28 kDa	Transcription Factor	100%	4	24.20%	0.08%	17
MHC class II regulatory factor RFX1 OS=Homo sapiens GN=RFX1 PE=1 SV=1	RFX1_HUMAN	105 kDa	Transcription Factor	100%	5	10.70%	0.08%	16
ETS-related transcription factor Elf-2 OS=Homo sapiens GN=ELF2 PE=1 SV=2	ELF2_HUMAN	64 kDa	Transcription Factor	100%	3	9.61%	0.08%	16
Glucocorticoid modulatory element-binding protein 2 OS=Homo sapiens GN=GMEB2 PE=1	GMEB2_HUMAN	56 kDa	Transcription Factor	100%	2	7.55%	0.08%	16
Upstream stimulatory factor 1 OS=Homo sapiens GN=USF1 PE=1 SV=1	USF1_HUMAN	34 kDa	Transcription Factor	100%	3	10.00%	0.07%	14
Transcription factor BTF3 OS=Homo sapiens GN=BTF3 PE=1 SV=1	BTF3_HUMAN	22 kDa	Transcription Factor	100%	3	33.50%	0.07%	14
SNW domain-containing protein 1 OS=Homo sapiens GN=SNW1 PE=1 SV=1	SNW1_HUMAN	61 kDa	Transcription Factor	100%	2	8.21%	0.06%	13
WD repeat-containing protein 61 OS=Homo sapiens GN=WDR61 PE=1 SV=1	WDR61_HUMAN	34 kDa	Transcription Factor	100%	2	16.10%	0.06%	13
CREB-binding protein OS=Homo sapiens GN=CREBBP PE=1 SV=3	CBP_HUMAN	265 kDa	Transcription Factor	100%	2	1.56%	0.06%	12
Krueppel-like factor 5 OS=Homo sapiens GN=KLF5 PE=1 SV=2	KLF5_HUMAN	51 kDa	Transcription Factor	100%	2	11.60%	0.06%	12
POU domain, class 2, transcription factor 1 OS=Homo sapiens GN=POU2F1 PE=1 SV=2	PO2F1_HUMAN	76 kDa	Transcription Factor	100%	3	9.96%	0.05%	11
Forkhead box protein P1 OS=Homo sapiens GN=FOXP1 PE=1 SV=1	FOXP1_HUMAN	75 kDa	Transcription Factor	100%	2	7.09%	0.05%	11
General transcription factor II-I OS=Homo sapiens GN=GTF2I PE=1 SV=2	GTF2I_HUMAN	112 kDa	Transcription Factor	100%	2	4.21%	0.05%	11
TAR DNA-binding protein 43 OS=Homo sapiens GN=TARDBP PE=1 SV=1	TADBP_HUMAN	45 kDa	Transcription Factor	100%	2	8.70%	0.05%	10
Nucleolar transcription factor 1 OS=Homo sapiens GN=UBTF PE=1 SV=1	UBF1_HUMAN	89 kDa	Transcription Factor	100%	2	3.66%	0.05%	10
Far upstream element-binding protein 1 OS=Homo sapiens GN=FUBP1 PE=1 SV=3	FUBP1_HUMAN	68 kDa	Transcription Factor	100%	2	5.75%	0.04%	9
Serine/threonine-protein phosphatase PP1-alpha catalytic subunit OS=Homo sapiens GN=F	PP1A_HUMAN (+2)	38 kDa	Transcription Factor	100%	2	9.39%	0.04%	9
Zinc finger protein 131 OS=Homo sapiens GN=ZNF131 PE=2 SV=2	ZN131_HUMAN	71 kDa	Transcription Factor	100%	2	6.74%	0.04%	9
Msx2-interacting protein OS=Homo sapiens GN=SPEN PE=1 SV=1	MINT_HUMAN	402 kDa	Transcription Factor	100%	3	1.56%	0.04%	8
Serine/threonine-protein phosphatase 2A 65 kDa regulatory subunit A alpha isoform OS=H	2AAA_HUMAN	65 kDa	Transcription Factor	100%	2	5.77%	0.04%	8
C-Myc-binding protein OS=Homo sapiens GN=MYCBP PE=1 SV=3	MYCBP_HUMAN	12 kDa	Transcription Factor	100%	2	35.90%	0.04%	8
Serine/threonine-protein phosphatase 2B catalytic subunit alpha isoform OS=Homo sapien	PP2BA_HUMAN	59 kDa	Transcription Factor	100%	2	8.25%	0.04%	8
Zinc finger and BTB domain-containing protein 3 OS=Homo sapiens GN=ZBTB3 PE=2 SV=1	ZBTB3_HUMAN	62 kDa	Transcription Factor	100%	2	10.50%	0.04%	8
Zinc finger BED domain-containing protein 1 OS=Homo sapiens GN=ZBED1 PE=1 SV=1	ZBED1_HUMAN	78 kDa	Transcription Factor	100%	2	4.32%	0.03%	7
Luc7-like protein 3 OS=Homo sapiens GN=LUC7L3 PE=1 SV=2	O95232|LC7L3_HUMAN	?	Transcription Factor	100%	2	0.00%	0.03%	6
Zinc finger MYM-type protein 2 OS=Homo sapiens GN=ZMYM2 PE=1 SV=1	ZMYM2_HUMAN	155 kDa	Transcription Factor	100%	2	2.54%	0.02%	5
Nuclear factor interleukin-3-regulated protein OS=Homo sapiens GN=NFIL3 PE=1 SV=2	NFIL3_HUMAN	51 kDa	Transcription Factor	100%	2	12.80%	0.01%	3
Zinc finger and BTB domain-containing protein 40 OS=Homo sapiens GN=ZBTB40 PE=1 SV=	ZBT40_HUMAN	138 kDa	Transcription Factor	99%	2	3.79%	0.03%	6
Activated RNA polymerase II transcriptional coactivator p15 OS=Homo sapiens GN=SUB1 P	TCP4_HUMAN	14 kDa	Transcription Factor	99%	2	21.30%	0.01%	3
Zinc finger and BTB domain-containing protein 7A OS=Homo sapiens GN=ZBTB7A PE=1 SV=	ZBT7A_HUMAN	61 kDa	Transcription Factor	99%	2	6.85%	0.01%	3
DNA-directed RNA polymerases I and III subunit RPAC1 OS=Homo sapiens GN=POLR1C PE=	RPAC1_HUMAN	39 kDa	Transcription Machine	100%	6	33.50%	0.18%	38
DNA-directed RNA polymerase III subunit RPC9 OS=Homo sapiens GN=CRCP PE=1 SV=1	RPC9_HUMAN	17 kDa	Transcription Machine	100%	2	31.10%	0.11%	24
DNA-directed RNA polymerase III subunit RPC1 OS=Homo sapiens GN=POLR3A PE=1 SV=2	RPC1_HUMAN	156 kDa	Transcription Machine	100%	5	6.62%	0.10%	22
Transcription initiation factor TFIID subunit 8 OS=Homo sapiens GN=TAF8 PE=1 SV=1	TAF8_HUMAN	34 kDa	Transcription Machine	100%	3	17.70%	0.09%	20
DNA-directed RNA polymerases I, II, and III subunit RPABC1 OS=Homo sapiens GN=POLR2E	RPAB1_HUMAN	25 kDa	Transcription Machine	100%	2	21.00%	0.07%	15
DNA-directed RNA polymerase III subunit RPC4 OS=Homo sapiens GN=POLR3D PE=1 SV=2	RPC4_HUMAN	44 kDa	Transcription Machine	100%	3	14.60%	0.06%	13
DNA-directed RNA polymerase III subunit RPC10 OS=Homo sapiens GN=POLR3K PE=1 SV=2	RPC10_HUMAN	12 kDa	Transcription Machine	100%	2	41.70%	0.06%	13
DNA-directed RNA polymerase III subunit RPC8 OS=Homo sapiens GN=POLR3H PE=1 SV=1	RPC8_HUMAN	23 kDa	Transcription Machine	100%	2	21.60%	0.06%	13
Mediator of RNA polymerase II transcription subunit 10 OS=Homo sapiens GN=MED10 PE=	MED10_HUMAN	16 kDa	Transcription Machine	100%	2	24.40%	0.05%	10
Transcription initiation factor TFIID subunit 5 OS=Homo sapiens GN=TAF5 PE=1 SV=3	TAF5_HUMAN	87 kDa	Transcription Machine	100%	2	8.50%	0.05%	10
Casein kinase II subunit alpha OS=Homo sapiens GN=CSNK2A1 PE=1 SV=1	CSK21_HUMAN	45 kDa	Transcription Machine	100%	3	9.46%	0.03%	7
Mediator of RNA polymerase II transcription subunit 1 OS=Homo sapiens GN=MED1 PE=1 S	MED1_HUMAN	168 kDa	Transcription Machine	100%	2	1.52%	0.03%	7
Transcription initiation factor TFIID subunit 10 OS=Homo sapiens GN=TAF10 PE=1 SV=1	TAF10_HUMAN	22 kDa	Transcription Machine	100%	2	15.60%	0.02%	5
RNA polymerase II-associated factor 1 homolog OS=Homo sapiens GN=PAF1 PE=1 SV=2	PAF1_HUMAN	60 kDa	Transcription Machine	100%	2	6.78%	0.02%	4
DNA-directed RNA polymerases I and III subunit RPAC2 OS=Homo sapiens GN=POLR1D PE=	RPAC2_HUMAN	15 kDa	Transcription Machine	99%	2	25.60%	0.04%	8

Proteins Below 95 % Threshold

Beta-actin-like protein 2 OS=Homo sapiens GN=ACTBL2 PE=1 SV=2	ACTBL_HUMAN	42 kDa		74%	1	17.30%	0.09%	18
Caprin-1 OS=Homo sapiens GN=CAPRIN1 PE=1 SV=2	CAPR1_HUMAN	78 kDa		74%	1	5.92%	0.08%	16
DNA polymerase epsilon subunit 3 OS=Homo sapiens GN=POLE3 PE=1 SV=1	DPOE3_HUMAN	17 kDa		74%	1	12.20%	0.08%	16
Chromosome transmission fidelity protein 8 homolog OS=Homo sapiens GN=CHTF8 PE=1 S	CTF8_HUMAN	13 kDa		74%	1	15.70%	0.07%	14
Importin subunit alpha-4 OS=Homo sapiens GN=KPNA4 PE=1 SV=1	IMA4_HUMAN	58 kDa		74%	1	3.26%	0.07%	14
Kinetochore-associated protein NSL1 homolog OS=Homo sapiens GN=NSL1 PE=1 SV=3	NSL1_HUMAN	32 kDa		74%	1	5.69%	0.06%	12
Transcription factor Sp3 OS=Homo sapiens GN=SP3 PE=1 SV=3	SP3_HUMAN	82 kDa		74%	1	3.59%	0.06%	12
ADP-ribosylation factor-like protein 1 OS=Homo sapiens GN=ARL1 PE=1 SV=1	ARL1_HUMAN	20 kDa		74%	1	8.84%	0.05%	11
DNA mismatch repair protein Msh2 OS=Homo sapiens GN=MSH2 PE=1 SV=1	MSH2_HUMAN	105 kDa		74%	1	1.50%	0.05%	11
Aryl hydrocarbon receptor nuclear translocator OS=Homo sapiens GN=ARNT PE=1 SV=1	ARNT_HUMAN	87 kDa		74%	1	3.55%	0.05%	10
E3 ubiquitin-protein ligase BRE1A OS=Homo sapiens GN=RNF20 PE=1 SV=2	BRE1A_HUMAN	114 kDa		74%	1	1.74%	0.05%	10
Cyclin-dependent kinase 2-associated protein 1 OS=Homo sapiens GN=CDK2AP1 PE=1 SV=1	CDKA1_HUMAN	12 kDa		74%	1	11.30%	0.05%	10
La-related protein 7 OS=Homo sapiens GN=LARP7 PE=1 SV=1	LARP7_HUMAN	67 kDa		74%	1	4.12%	0.05%	10
Homeobox protein Meis2 OS=Homo sapiens GN=MEIS2 PE=1 SV=2	MEIS2_HUMAN	52 kDa		74%	1	13.60%	0.05%	10
Brain acid soluble protein 1 OS=Homo sapiens GN=BASP1 PE=1 SV=2	P80723|BASP1_HUMAN	?		74%	1	0.00%	0.05%	10
Septin-9 OS=Homo sapiens GN=SEPT9 PE=1 SV=2	SEPT9_HUMAN	65 kDa		74%	1	3.07%	0.05%	10
Structural maintenance of chromosomes protein 1A OS=Homo sapiens GN=SMC1A PE=1 S\	SMC1A_HUMAN	143 kDa		74%	1	1.14%	0.05%	10
Homeobox protein cut-like 1 OS=Homo sapiens GN=CUX1 PE=1 SV=2	CUX1_HUMAN	164 kDa		74%	1	2.13%	0.04%	9
RNA-binding protein FUS OS=Homo sapiens GN=FUS PE=1 SV=1	FUS_HUMAN	53 kDa		74%	1	6.08%	0.04%	9
DNA-directed RNA polymerase II subunit RPB3 OS=Homo sapiens GN=POLR2C PE=1 SV=2	RPB3_HUMAN	31 kDa		74%	1	8.36%	0.04%	9
DNA-directed RNA polymerase III subunit RPC5 OS=Homo sapiens GN=POLR3E PE=1 SV=1	RPC5_HUMAN	80 kDa		74%	1	4.66%	0.04%	9
Hcp beta-lactamase-like protein C1orf163 OS=Homo sapiens GN=C1orf163 PE=1 SV=1	CA163_HUMAN	26 kDa		74%	1	11.70%	0.04%	8
ETS-related transcription factor Elf-3 OS=Homo sapiens GN=ELF3 PE=1 SV=1	ELF3_HUMAN	41 kDa		74%	1	5.12%	0.04%	8
LIM and senescent cell antigen-like-containing domain protein 1 OS=Homo sapiens GN=LIN	LIMS1_HUMAN	37 kDa		74%	1	4.62%	0.04%	8
Mediator of RNA polymerase II transcription subunit 11 OS=Homo sapiens GN=MED11 PE=	MED11_HUMAN	13 kDa		74%	1	15.40%	0.04%	8
~~Myosin light polypeptide 6 OS=Homo sapiens GN=MYL6 PE=1 SV=2~~	MYL6_HUMAN	17 kDa		74%	1	10.60%	0.04%	8
Pre-mRNA-processing-splicing factor 8 OS=Homo sapiens GN=PRPF8 PE=1 SV=2	PRP8_HUMAN	274 kDa		74%	1	0.86%	0.04%	8
Heterogeneous nuclear ribonucleoprotein A/B OS=Homo sapiens GN=HNRNPAB PE=1 SV=2	ROAA_HUMAN	36 kDa		74%	1	5.72%	0.04%	8
Small nuclear ribonucleoprotein E OS=Homo sapiens GN=SNRPE PE=1 SV=1	RUXE_HUMAN	11 kDa		74%	1	27.20%	0.04%	8
Protein S100-A10 OS=Homo sapiens GN=S100A10 PE=1 SV=2	S10AA_HUMAN	11 kDa		74%	1	17.50%	0.04%	8
Superkiller viralicidic activity 2-like 2 OS=Homo sapiens GN=SKIV2L2 PE=1 SV=3	SK2L2_HUMAN	118 kDa		74%	1	2.02%	0.04%	8
Small nuclear ribonucleoprotein Sm D1 OS=Homo sapiens GN=SNRPD1 PE=1 SV=1	SMD1_HUMAN	13 kDa		74%	1	16.80%	0.04%	8
Ubiquilin-4 OS=Homo sapiens GN=UBQLN4 PE=1 SV=2	UBQL4_HUMAN	64 kDa		74%	1	8.15%	0.04%	8
Vasodilator-stimulated phosphoprotein OS=Homo sapiens GN=VASP PE=1 SV=3	VASP_HUMAN	40 kDa		74%	1	6.58%	0.04%	8
YTH domain family protein 2 OS=Homo sapiens GN=YTHDF2 PE=1 SV=2	YTHD2_HUMAN	62 kDa		74%	1	5.87%	0.04%	8
Dual specificity mitogen-activated protein kinase kinase 1 OS=Homo sapiens GN=MAP2K1 I	MP2K1_HUMAN (+1)	43 kDa		74%	1	5.60%	0.03%	7
Nuclear transcription factor Y subunit beta OS=Homo sapiens GN=NFYB PE=1 SV=2	NFYB_HUMAN	23 kDa		74%	1	10.10%	0.03%	7
Prefoldin subunit 1 OS=Homo sapiens GN=PFDN1 PE=1 SV=2	PFD1_HUMAN	14 kDa		74%	1	19.70%	0.03%	7
Protein BAT2-like 2 OS=Homo sapiens GN=BAT2L2 PE=1 SV=2	Q9Y520|BA2L2_HUMAN	?		74%	1	0.00%	0.03%	7
HLA class I histocompatibility antigen, A-1 alpha chain OS=Homo sapiens GN=HLA-A PE=1 S	1A01_HUMAN (+31)	41 kDa		74%	1	6.58%	0.03%	6
A-kinase anchor protein 8 OS=Homo sapiens GN=AKAP8 PE=1 SV=1	AKAP8_HUMAN	76 kDa		74%	1	2.89%	0.03%	6
ADP-ribosylation factor 1 OS=Homo sapiens GN=ARF1 PE=1 SV=2	ARF1_HUMAN (+1)	21 kDa		74%	1	19.90%	0.03%	6
Chromobox protein homolog 1 OS=Homo sapiens GN=CBX1 PE=1 SV=1	CBX1_HUMAN (+1)	21 kDa		74%	1	8.65%	0.03%	6
Cellular nucleic acid-binding protein OS=Homo sapiens GN=CNBP PE=1 SV=1	CNBP_HUMAN	19 kDa		74%	1	8.47%	0.03%	6
COMM domain-containing protein 9 OS=Homo sapiens GN=COMMD9 PE=1 SV=2	COMD9_HUMAN	22 kDa		74%	1	12.60%	0.03%	6
ATP-dependent RNA helicase DDX19B OS=Homo sapiens GN=DDX19B PE=1 SV=1	DD19B_HUMAN	54 kDa		74%	1	29.60%	0.03%	6
Alpha-enolase OS=Homo sapiens GN=ENO1 PE=1 SV=2	ENOA_HUMAN (+2)	47 kDa		74%	1	5.07%	0.03%	6
Enhancer of rudimentary homolog OS=Homo sapiens GN=ERH PE=1 SV=1	ERH_HUMAN	12 kDa		74%	1	16.30%	0.03%	6
Transcription factor ETV6 OS=Homo sapiens GN=ETV6 PE=1 SV=1	ETV6_HUMAN	53 kDa		74%	1	4.65%	0.03%	6
BTB/POZ domain-containing protein KCTD3 OS=Homo sapiens GN=KCTD3 PE=1 SV=2	KCTD3_HUMAN	89 kDa		74%	1	2.82%	0.03%	6
Kinesin-like protein KIF23 OS=Homo sapiens GN=KIF23 PE=1 SV=3	KIF23_HUMAN	110 kDa		74%	1	1.88%	0.03%	6
Pyruvate kinase isozymes M1/M2 OS=Homo sapiens GN=PKM2 PE=1 SV=4	KPYM_HUMAN	58 kDa		74%	1	3.01%	0.03%	6
Leucine-rich repeat and WD repeat-containing protein 1 OS=Homo sapiens GN=LRWD1 PE=	LRWD1_HUMAN	71 kDa		74%	1	2.63%	0.03%	6
Nuclear pore glycoprotein p62 OS=Homo sapiens GN=NUP62 PE=1 SV=3	NUP62_HUMAN	53 kDa		74%	1	2.68%	0.03%	6
DDB1- and CUL4-associated factor 7 OS=Homo sapiens GN=DCAF7 PE=1 SV=1	P61962|DCAF7_HUMAN	?		74%	1	0.00%	0.03%	6
Transcriptional repressor p66-beta OS=Homo sapiens GN=GATAD2B PE=1 SV=1	P66B_HUMAN	65 kDa		74%	1	4.22%	0.03%	6
Polyadenylate-binding protein 1 OS=Homo sapiens GN=PABPC1 PE=1 SV=2	PABP1_HUMAN (+1)	71 kDa		74%	1	3.77%	0.03%	6
Choline-phosphate cytidylyltransferase A OS=Homo sapiens GN=PCYT1A PE=1 SV=2	PCY1A_HUMAN	42 kDa		74%	1	4.63%	0.03%	6
Peptidyl-prolyl cis-trans isomerase-like 3 OS=Homo sapiens GN=PPIL3 PE=1 SV=1	PPIL3_HUMAN	18 kDa		74%	1	11.80%	0.03%	6
Proline-rich protein PRCC OS=Homo sapiens GN=PRCC PE=1 SV=1	PRCC_HUMAN	52 kDa		74%	1	4.28%	0.03%	6
26S protease regulatory subunit 6B OS=Homo sapiens GN=PSMC4 PE=1 SV=2	PRS6B_HUMAN	47 kDa		74%	1	4.31%	0.03%	6
Cell cycle regulator Mat89Bb homolog OS=Homo sapiens GN=C12orf11 PE=1 SV=2	Q9NVM9|M89BB_HUMAN	?		74%	1	0.00%	0.03%	6
Histone-binding protein RBBP7 OS=Homo sapiens GN=RBBP7 PE=1 SV=1	RBBP7_HUMAN	48 kDa		74%	1	8.24%	0.03%	6
Protein S100-A16 OS=Homo sapiens GN=S100A16 PE=1 SV=1	S10AG_HUMAN	12 kDa		74%	1	10.70%	0.03%	6
SWI/SNF complex subunit SMARCC2 OS=Homo sapiens GN=SMARCC2 PE=1 SV=1	SMRC2_HUMAN	133 kDa		74%	1	3.71%	0.03%	6
Signal recognition particle 14 kDa protein OS=Homo sapiens GN=SRP14 PE=1 SV=2	SRP14_HUMAN	15 kDa		74%	1	21.30%	0.03%	6
Signal transducer and activator of transcription 3 OS=Homo sapiens GN=STAT3 PE=1 SV=2	STAT3_HUMAN	88 kDa		74%	1	3.38%	0.03%	6
Transcription initiation factor TFIID subunit 4 OS=Homo sapiens GN=TAF4 PE=1 SV=2	TAF4_HUMAN	110 kDa		74%	1	2.76%	0.03%	6
AN1-type zinc finger protein 6 OS=Homo sapiens GN=ZFAND6 PE=2 SV=2	ZFAN6_HUMAN	23 kDa		74%	1	12.00%	0.03%	6
Zinc finger protein 148 OS=Homo sapiens GN=ZNF148 PE=1 SV=2	ZN148_HUMAN	89 kDa		74%	1	2.27%	0.03%	6
Zinc finger protein 629 OS=Homo sapiens GN=ZNF629 PE=2 SV=2	ZN629_HUMAN	97 kDa		74%	1	5.64%	0.03%	6
Zyxin OS=Homo sapiens GN=ZYX PE=1 SV=1	ZYX_HUMAN	61 kDa		74%	1	3.67%	0.03%	6
Cleavage and polyadenylation specificity factor subunit 3 OS=Homo sapiens GN=CPSF3 PE=	CPSF3_HUMAN	77 kDa		74%	1	2.49%	0.02%	5
Dynein light chain Tctex-type 1 OS=Homo sapiens GN=DYNLT1 PE=1 SV=1	DYLT1_HUMAN	12 kDa		74%	1	15.90%	0.02%	5
Transcription elongation factor B polypeptide 1 OS=Homo sapiens GN=TCEB1 PE=1 SV=1	ELOC_HUMAN	12 kDa		74%	1	17.90%	0.02%	5
Fidgetin-like protein 1 OS=Homo sapiens GN=FIGNL1 PE=1 SV=2	FIGL1_HUMAN	74 kDa		74%	1	2.82%	0.02%	5
Heat shock 70 kDa protein 4 OS=Homo sapiens GN=HSPA4 PE=1 SV=4	HSP74_HUMAN	94 kDa		74%	1	4.05%	0.02%	5
Nuclear receptor coactivator 3 OS=Homo sapiens GN=NCOA3 PE=1 SV=1	NCOA3_HUMAN	155 kDa		74%	1	1.62%	0.02%	5
Alpha-parvin OS=Homo sapiens GN=PARVA PE=1 SV=1	PARVA_HUMAN	42 kDa		74%	1	3.76%	0.02%	5
Pre-mRNA-processing factor 40 homolog A OS=Homo sapiens GN=PRPF40A PE=1 SV=2	PR40A_HUMAN	109 kDa		74%	1	1.57%	0.02%	5
Q9UPA5|BSN_HUMAN-R	Q9UPA5|BSN_HUMAN-R	?		74%	1	0.00%	0.02%	5
Replication factor C subunit 3 OS=Homo sapiens GN=RFC3 PE=1 SV=2	RFC3_HUMAN	41 kDa		74%	1	5.06%	0.02%	5
DNA-directed RNA polymerases I, II, and III subunit RPABC3 OS=Homo sapiens GN=POLR2H	RPAB3_HUMAN	17 kDa		74%	1	18.00%	0.02%	5
Protein S100-A11 OS=Homo sapiens GN=S100A11 PE=1 SV=2	S10AB_HUMAN	12 kDa		74%	1	15.20%	0.02%	5
Sodium channel modifier 1 OS=Homo sapiens GN=SCNM1 PE=1 SV=1	SCNM1_HUMAN	26 kDa		74%	1	11.30%	0.02%	5
Splicing factor 3B subunit 3 OS=Homo sapiens GN=SF3B3 PE=1 SV=4	SF3B3_HUMAN	136 kDa		74%	1	1.97%	0.02%	5
Phosphatidylinositol-3,4,5-trisphosphate 5-phosphatase 2 OS=Homo sapiens GN=INPPL1 PI	SHIP2_HUMAN	139 kDa		74%	1	1.11%	0.02%	5
Transformation/transcription domain-associated protein OS=Homo sapiens GN=TRRAP PE=	TRRAP_HUMAN	438 kDa		74%	1	0.70%	0.02%	5
Spliceosome RNA helicase BAT1 OS=Homo sapiens GN=BAT1 PE=1 SV=1	UAP56_HUMAN	49 kDa		74%	1	22.70%	0.02%	5
Zinc finger protein 687 OS=Homo sapiens GN=ZNF687 PE=1 SV=1	ZN687_HUMAN	130 kDa		74%	1	2.18%	0.02%	5
Amino-terminal enhancer of split OS=Homo sapiens GN=AES PE=1 SV=4	AES_HUMAN	22 kDa		74%	1	13.20%	0.02%	4
Fructose-bisphosphate aldolase A OS=Homo sapiens GN=ALDOA PE=1 SV=2	ALDOA_HUMAN	39 kDa		74%	1	5.49%	0.02%	4
Ankyrin repeat domain-containing protein 11 OS=Homo sapiens GN=ANKRD11 PE=1 SV=1	ANR11_HUMAN	298 kDa		74%	1	1.01%	0.02%	4
Serine-protein kinase ATM OS=Homo sapiens GN=ATM PE=1 SV=1	ATM_HUMAN	351 kDa		74%	1	0.65%	0.02%	4
Calmodulin OS=Homo sapiens GN=CALM1 PE=1 SV=2	CALM_HUMAN	17 kDa		74%	1	10.70%	0.02%	4
Anaphase-promoting complex subunit CDC26 OS=Homo sapiens GN=CDC26 PE=1 SV=1	CDC26_HUMAN	10 kDa		74%	1	28.20%	0.02%	4
Chromatin accessibility complex protein 1 OS=Homo sapiens GN=CHRAC1 PE=2 SV=1	CHRC1_HUMAN	15 kDa		74%	1	13.70%	0.02%	4
Cleavage and polyadenylation specificity factor subunit 5 OS=Homo sapiens GN=NUDT21 P	CPSF5_HUMAN	26 kDa		74%	1	7.93%	0.02%	4
ATP-dependent RNA helicase A OS=Homo sapiens GN=DHX9 PE=1 SV=4	DHX9_HUMAN	141 kDa		74%	1	0.95%	0.02%	4
ETS-related transcription factor Elf-1 OS=Homo sapiens GN=ELF1 PE=1 SV=2	ELF1_HUMAN	67 kDa		74%	1	2.42%	0.02%	4
Golgin subfamily A member 1 OS=Homo sapiens GN=GOLGA1 PE=1 SV=2	GOGA1_HUMAN	88 kDa		74%	1	2.22%	0.02%	4
Histone deacetylase 3 OS=Homo sapiens GN=HDAC3 PE=1 SV=2	HDAC3_HUMAN	49 kDa		74%	1	2.80%	0.02%	4
Protein HEXIM1 OS=Homo sapiens GN=HEXIM1 PE=1 SV=1	HEXI1_HUMAN	41 kDa		74%	1	3.90%	0.02%	4
Eukaryotic translation initiation factor 2 subunit 1 OS=Homo sapiens GN=EIF2S1 PE=1 SV=3	IF2A_HUMAN	36 kDa		74%	1	4.76%	0.02%	4
Immunoglobulin-binding protein 1 OS=Homo sapiens GN=IGBP1 PE=1 SV=1	IGBP1_HUMAN	39 kDa		74%	1	5.01%	0.02%	4
Integrator complex subunit 9 OS=Homo sapiens GN=INTS9 PE=1 SV=2	INT9_HUMAN	74 kDa		74%	1	1.82%	0.02%	4
Ras GTPase-activating-like protein IQGAP1 OS=Homo sapiens GN=IQGAP1 PE=1 SV=1	IQGA1_HUMAN	189 kDa		74%	1	1.15%	0.02%	4
IWS1 homolog OS=Homo sapiens GN=IWS1 PE=1 SV=2	IWS1_HUMAN	92 kDa		74%	1	2.32%	0.02%	4
Uncharacterized protein KIAA1267 OS=Homo sapiens GN=KIAA1267 PE=1 SV=1	K1267_HUMAN	121 kDa		74%	1	2.17%	0.02%	4
Calcium/calmodulin-dependent protein kinase type II gamma chain OS=Homo sapiens GN=	KCC2G_HUMAN	63 kDa		74%	1	4.48%	0.02%	4
LIM domain only protein 7 OS=Homo sapiens GN=LMO7 PE=1 SV=2	LMO7_HUMAN	193 kDa		74%	1	0.95%	0.02%	4
Leucine-rich repeat-containing protein 47 OS=Homo sapiens GN=LRRC47 PE=1 SV=1	LRC47_HUMAN	63 kDa		74%	1	3.09%	0.02%	4
Mortality factor 4-like protein 2 OS=Homo sapiens GN=MORF4L2 PE=1 SV=1	MO4L2_HUMAN	32 kDa		74%	1	6.25%	0.02%	4
Dual specificity mitogen-activated protein kinase kinase 3 OS=Homo sapiens GN=MAP2K3 I	MP2K3_HUMAN	39 kDa		74%	1	5.19%	0.02%	4
DNA mismatch repair protein Msh6 OS=Homo sapiens GN=MSH6 PE=1 SV=2	MSH6_HUMAN	153 kDa		74%	1	0.88%	0.02%	4
Mucin-1 OS=Homo sapiens GN=MUC1 PE=1 SV=2	MUC1_HUMAN	122 kDa		74%	1	0.88%	0.02%	4
Nuclear distribution protein nudE homolog 1 OS=Homo sapiens GN=NDE1 PE=1 SV=2	NDE1_HUMAN (+1)	39 kDa		74%	1	3.47%	0.02%	4
Origin recognition complex subunit 3 OS=Homo sapiens GN=ORC3L PE=1 SV=1	ORC3_HUMAN	82 kDa		74%	1	1.69%	0.02%	4
Prefoldin subunit 4 OS=Homo sapiens GN=PFDN4 PE=1 SV=1	PFD4_HUMAN	15 kDa		74%	1	8.96%	0.02%	4
Homeobox protein PKNOX1 OS=Homo sapiens GN=PKNOX1 PE=1 SV=3	PKNX1_HUMAN	48 kDa		74%	1	5.73%	0.02%	4
Pre-mRNA branch site protein p14 OS=Homo sapiens GN=SF3B14 PE=1 SV=1	PM14_HUMAN	15 kDa		74%	1	9.60%	0.02%	4
Mismatch repair endonuclease PMS2 OS=Homo sapiens GN=PMS2 PE=1 SV=1	PMS2_HUMAN	96 kDa		74%	1	1.97%	0.02%	4
Ribose-phosphate pyrophosphokinase 1 OS=Homo sapiens GN=PRPS1 PE=1 SV=2	PRPS1_HUMAN (+1)	35 kDa		74%	1	4.72%	0.02%	4
E3 ubiquitin-protein ligase RAD18 OS=Homo sapiens GN=RAD18 PE=1 SV=1	RAD18_HUMAN	56 kDa		74%	1	2.83%	0.02%	4
Ras-related protein Rap-1A OS=Homo sapiens GN=RAP1A PE=1 SV=1	RAP1A_HUMAN (+2)	21 kDa		74%	1	6.52%	0.02%	4
Atherin OS=Homo sapiens GN=SAMD1 PE=1 SV=1	SAMD1_HUMAN	56 kDa		74%	1	7.43%	0.02%	4
SAPS domain family member 3 OS=Homo sapiens GN=SAPS3 PE=1 SV=2	SAPS3_HUMAN	98 kDa		74%	1	1.60%	0.02%	4
SAGA-associated factor 29 homolog OS=Homo sapiens GN=CCDC101 PE=2 SV=1	SGF29_HUMAN	33 kDa		74%	1	4.44%	0.02%	4
SH3KBP1-binding protein 1 OS=Homo sapiens GN=SHKBP1 PE=2 SV=2	SHKB1_HUMAN	76 kDa		74%	1	3.25%	0.02%	4
Structural maintenance of chromosomes protein 2 OS=Homo sapiens GN=SMC2 PE=1 SV=1	SMC2_HUMAN	136 kDa		74%	1	1.67%	0.02%	4
SSXT protein OS=Homo sapiens GN=SS18 PE=2 SV=3	SSXT_HUMAN	46 kDa		74%	1	4.31%	0.02%	4
Transcription initiation factor TFIID subunit 9B OS=Homo sapiens GN=TAF9B PE=1 SV=1	TAF9B_HUMAN (+1)	28 kDa		74%	1	4.38%	0.02%	4
Tubulin alpha chain-like 3 OS=Homo sapiens GN=TUBAL3 PE=1 SV=2	TBAL3_HUMAN	50 kDa		74%	1	3.81%	0.02%	4
T-complex protein 1 subunit alpha OS=Homo sapiens GN=CCT1 PE=1 SV=1	TCPA_HUMAN	60 kDa		74%	1	3.24%	0.02%	4
Telomeric repeat-binding factor 2-interacting protein 1 OS=Homo sapiens GN=TERF2IP PE=	TE2IP_HUMAN	44 kDa		74%	1	7.27%	0.02%	4
Transitional endoplasmic reticulum ATPase OS=Homo sapiens GN=VCP PE=1 SV=4	TERA_HUMAN	89 kDa		74%	1	2.11%	0.02%	4
Mitochondrial import inner membrane translocase subunit Tim8 A OS=Homo sapiens GN=T	TIM8A_HUMAN	11 kDa		74%	1	11.30%	0.02%	4
TOX high mobility group box family member 4 OS=Homo sapiens GN=TOX4 PE=1 SV=1	TOX4_HUMAN	66 kDa		74%	1	1.61%	0.02%	4
Dual specificity protein kinase TTK OS=Homo sapiens GN=TTK PE=1 SV=2	TTK_HUMAN	97 kDa		74%	1	1.52%	0.02%	4
Zinc finger protein with KRAB and SCAN domains 3 OS=Homo sapiens GN=ZKSCAN3 PE=1 S	ZKSC3_HUMAN (+1)	61 kDa		74%	1	3.53%	0.02%	4
Zinc finger protein 281 OS=Homo sapiens GN=ZNF281 PE=1 SV=1	ZN281_HUMAN	97 kDa		74%	1	3.24%	0.02%	4
Zinc finger protein 335 OS=Homo sapiens GN=ZNF335 PE=1 SV=1	ZN335_HUMAN	145 kDa		74%	1	2.09%	0.02%	4
Zinc finger protein 784 OS=Homo sapiens GN=ZNF784 PE=2 SV=1	ZN784_HUMAN	34 kDa		74%	1	12.10%	0.02%	4
HMG box transcription factor BBX OS=Homo sapiens GN=BBX PE=2 SV=1	BBX_HUMAN	105 kDa		74%	1	2.02%	0.01%	3
UPF0363 protein C7orf20 OS=Homo sapiens GN=C7orf20 PE=1 SV=1	CG020_HUMAN	37 kDa		74%	1	4.59%	0.01%	3
Coiled-coil-helix-coiled-coil-helix domain-containing protein 2, mitochondrial OS=Homo sa	CHCH2_HUMAN (+1)	16 kDa		74%	1	18.50%	0.01%	3
Casein kinase II subunit beta OS=Homo sapiens GN=CSNK2B PE=1 SV=1	CSK2B_HUMAN	25 kDa		74%	1	7.44%	0.01%	3
Death domain-associated protein 6 OS=Homo sapiens GN=DAXX PE=1 SV=2	DAXX_HUMAN	81 kDa		74%	1	4.73%	0.01%	3
Probable ATP-dependent RNA helicase DDX17 OS=Homo sapiens GN=DDX17 PE=1 SV=1	DDX17_HUMAN	72 kDa		74%	1	3.54%	0.01%	3
Dynein light chain 2, cytoplasmic OS=Homo sapiens GN=DYNLL2 PE=1 SV=1	DYL2_HUMAN	10 kDa		74%	1	42.70%	0.01%	3
Enhancer of yellow 2 transcription factor homolog OS=Homo sapiens GN=ENY2 PE=2 SV=1	ENY2_HUMAN	12 kDa		74%	1	16.80%	0.01%	3
Steroid hormone receptor ERR1 OS=Homo sapiens GN=ESRRA PE=1 SV=2	ERR1_HUMAN	55 kDa		74%	1	2.12%	0.01%	3
Far upstream element-binding protein 2 OS=Homo sapiens GN=KHSRP PE=1 SV=3	FUBP2_HUMAN	73 kDa		74%	1	2.54%	0.01%	3
Guanine nucleotide-binding protein G(I)/G(S)/G(T) subunit beta-2 OS=Homo sapiens GN=G	GBB2_HUMAN	37 kDa		74%	1	13.80%	0.01%	3
GON-4-like protein OS=Homo sapiens GN=GON4L PE=1 SV=1	GON4L_HUMAN	249 kDa		74%	1	1.38%	0.01%	3
Hepatocyte growth factor-regulated tyrosine kinase substrate OS=Homo sapiens GN=HGS I	HGS_HUMAN	86 kDa		74%	1	2.19%	0.01%	3
Heterogeneous nuclear ribonucleoprotein H2 OS=Homo sapiens GN=HNRNPH2 PE=1 SV=1	HNRH2_HUMAN	49 kDa		74%	1	12.20%	0.01%	3
Integrator complex subunit 6 OS=Homo sapiens GN=INTS6 PE=1 SV=1	INT6_HUMAN	100 kDa		74%	1	1.69%	0.01%	3
Transcription factor jun-B OS=Homo sapiens GN=JUNB PE=1 SV=1	JUNB_HUMAN	36 kDa		74%	1	4.61%	0.01%	3
Keratin, type I cytoskeletal 16 OS=Homo sapiens GN=KRT16 PE=1 SV=4	K1C16_HUMAN	51 kDa		74%	1	3.81%	0.01%	3
Keratin, type I cytoskeletal 18 OS=Homo sapiens GN=KRT18 PE=1 SV=2	K1C18_HUMAN	48 kDa		74%	1	4.19%	0.01%	3
Mediator of RNA polymerase II transcription subunit 12 OS=Homo sapiens GN=MED12 PE=	MED12_HUMAN	243 kDa		74%	1	1.70%	0.01%	3
Mediator of RNA polymerase II transcription subunit 8 OS=Homo sapiens GN=MED8 PE=1 S	MED8_HUMAN	29 kDa		74%	1	6.34%	0.01%	3
MARCKS-related protein OS=Homo sapiens GN=MARCKSL1 PE=1 SV=2	MRP_HUMAN	20 kDa		74%	1	17.40%	0.01%	3
Nuclear transcription factor Y subunit gamma OS=Homo sapiens GN=NFYC PE=1 SV=3	NFYC_HUMAN	50 kDa		74%	1	2.84%	0.01%	3
Lysine-specific demethylase 4B OS=Homo sapiens GN=KDM4B PE=1 SV=4	O94953|KDM4B_HUMAN	?		74%	1	0.00%	0.01%	3
Origin recognition complex subunit 1 OS=Homo sapiens GN=ORC1L PE=1 SV=2	ORC1_HUMAN	97 kDa		74%	1	2.44%	0.01%	3
High affinity cAMP-specific and IBMX-insensitive 3',5'-cyclic phosphodiesterase 8B OS=Hon	PDE8B_HUMAN	99 kDa		74%	1	2.82%	0.01%	3
Protein disulfide-isomerase A3 OS=Homo sapiens GN=PDIA3 PE=1 SV=4	PDIA3_HUMAN	57 kDa		74%	1	4.16%	0.01%	3
PHD finger protein 3 OS=Homo sapiens GN=PHF3 PE=1 SV=3	PHF3_HUMAN	229 kDa		74%	1	1.37%	0.01%	3
DDB1- and CUL4-associated factor 6 OS=Homo sapiens GN=DCAF6 PE=1 SV=1	Q58WW2|DCAF6_HUMAN	?		74%	1	0.00%	0.01%	3
60S ribosomal protein L22 OS=Homo sapiens GN=RPL22 PE=1 SV=2	RL22_HUMAN	15 kDa		74%	1	20.30%	0.01%	3
Heterogeneous nuclear ribonucleoprotein A3 OS=Homo sapiens GN=HNRNPA3 PE=1 SV=2	ROA3_HUMAN	40 kDa		74%	1	4.23%	0.01%	3
DNA-directed RNA polymerases I, II, and III subunit RPABC4 OS=Homo sapiens GN=POLR2K	RPAB4_HUMAN	7 kDa		74%	1	31.00%	0.01%	3
DNA-directed RNA polymerase III subunit RPC7 OS=Homo sapiens GN=POLR3G PE=1 SV=1	RPC7_HUMAN	27 kDa		74%	1	9.87%	0.01%	3

Regulation of nuclear pre-mRNA domain-containing protein 1B OS=Homo sapiens GN=RPR	RPR1B_HUMAN	37 kDa	74%	1	4.60%	0.01%	3
40S ribosomal protein S12 OS=Homo sapiens GN=RPS12 PE=1 SV=2	RS12_HUMAN	15 kDa	74%	1	0.00%	0.01%	3
Squamous cell carcinoma antigen recognized by T-cells 3 OS=Homo sapiens GN=SART3 PE=	SART3_HUMAN	110 kDa	74%	1	3.32%	0.01%	3
Pre-mRNA-splicing factor SLU7 OS=Homo sapiens GN=SLU7 PE=1 SV=1	SLU7_HUMAN	68 kDa	74%	1	4.61%	0.01%	3
TAF5-like RNA polymerase II p300/CBP-associated factor-associated factor 65 kDa subunit !	TAF5L_HUMAN	66 kDa	74%	1	3.40%	0.01%	3
Transducin-like enhancer protein 3 OS=Homo sapiens GN=TLE3 PE=1 SV=2	TLE3_HUMAN	83 kDa	74%	1	2.72%	0.01%	3
Vimentin OS=Homo sapiens GN=VIM PE=1 SV=4	VIME_HUMAN	54 kDa	74%	1	2.15%	0.01%	3
14-3-3 protein theta OS=Homo sapiens GN=YWHAQ PE=1 SV=1	1433T_HUMAN	28 kDa	74%	1	11.80%	0.01%	2
Activator of 90 kDa heat shock protein ATPase homolog 1 OS=Homo sapiens GN=AHSA1 PE	AHSA1_HUMAN	38 kDa	74%	1	6.51%	0.01%	2
BAG family molecular chaperone regulator 3 OS=Homo sapiens GN=BAG3 PE=1 SV=3	BAG3_HUMAN	62 kDa	74%	1	6.61%	0.01%	2
Uncharacterized protein C1orf112 OS=Homo sapiens GN=C1orf112 PE=2 SV=1	CA112_HUMAN	97 kDa	74%	1	2.23%	0.01%	2
Parafibromin OS=Homo sapiens GN=CDC73 PE=1 SV=1	CDC73_HUMAN	61 kDa	74%	1	3.77%	0.01%	2
CUG-BP- and ETR-3-like factor 1 OS=Homo sapiens GN=CUGBP1 PE=1 SV=2	CELF1_HUMAN	52 kDa	74%	1	3.29%	0.01%	2
Coatomer subunit beta' OS=Homo sapiens GN=COPB2 PE=1 SV=2	COPB2_HUMAN	102 kDa	74%	1	2.10%	0.01%	2
Cleavage and polyadenylation specificity factor subunit 7 OS=Homo sapiens GN=CPSF7 PE=	CPSF7_HUMAN	52 kDa	74%	1	5.31%	0.01%	2
Catenin alpha-1 OS=Homo sapiens GN=CTNNA1 PE=1 SV=1	CTNA1_HUMAN	100 kDa	74%	1	2.87%	0.01%	2
CpG-binding protein OS=Homo sapiens GN=CXXC1 PE=1 SV=2	CXXC1_HUMAN	76 kDa	74%	1	3.96%	0.01%	2
ETS domain-containing protein Elk-1 OS=Homo sapiens GN=ELK1 PE=1 SV=2	ELK1_HUMAN	45 kDa	74%	1	6.31%	0.01%	2
Histone acetyltransferase p300 OS=Homo sapiens GN=EP300 PE=1 SV=1	EP300_HUMAN	264 kDa	74%	1	1.74%	0.01%	2
Exosome component 10 OS=Homo sapiens GN=EXOSC10 PE=1 SV=2	EXOSX_HUMAN	101 kDa	74%	1	2.37%	0.01%	2
Fanconi anemia group G protein OS=Homo sapiens GN=FANCG PE=1 SV=1	FANCG_HUMAN	69 kDa	74%	1	3.54%	0.01%	2
Glycogen synthase kinase-3 beta OS=Homo sapiens GN=GSK3B PE=1 SV=2	GSK3B_HUMAN	47 kDa	74%	1	5.71%	0.01%	2
Hepatoma-derived growth factor OS=Homo sapiens GN=HDGF PE=1 SV=1	HDGF_HUMAN	27 kDa	74%	1	11.20%	0.01%	2
SWI/SNF-related matrix-associated actin-dependent regulator of chromatin subfamily E me	HM20B_HUMAN	36 kDa	74%	1	7.57%	0.01%	2
Importin subunit alpha-1 OS=Homo sapiens GN=KPNA1 PE=1 SV=2	IMA1_HUMAN	60 kDa	74%	1	3.90%	0.01%	2
Integrator complex subunit 1 OS=Homo sapiens GN=INTS1 PE=1 SV=2	INT1_HUMAN	244 kDa	74%	1	0.87%	0.01%	2
Kinesin-like protein KIF2C OS=Homo sapiens GN=KIF2C PE=1 SV=2	KIF2C_HUMAN	81 kDa	74%	1	3.17%	0.01%	2
La-related protein 4 OS=Homo sapiens GN=LARP4 PE=1 SV=3	LARP4_HUMAN	81 kDa	74%	1	1.80%	0.01%	2
Mediator of RNA polymerase II transcription subunit 15 OS=Homo sapiens GN=MED15 PE=	MED15_HUMAN	87 kDa	74%	1	2.16%	0.01%	2
Mediator of RNA polymerase II transcription subunit 7 OS=Homo sapiens GN=MED7 PE=1 S	MED7_HUMAN	27 kDa	74%	1	8.58%	0.01%	2
Mortality factor 4-like protein 1 OS=Homo sapiens GN=MORF4L1 PE=1 SV=2	MO4L1_HUMAN	41 kDa	74%	1	4.97%	0.01%	2
MORC family CW-type zinc finger protein 2 OS=Homo sapiens GN=MORC2 PE=1 SV=2	MORC2_HUMAN	118 kDa	74%	1	2.23%	0.01%	2
Nascent polypeptide-associated complex subunit alpha OS=Homo sapiens GN=NACA PE=1	NACA_HUMAN	23 kDa	74%	1	6.98%	0.01%	2
Probable ribosome biogenesis protein NEP1 OS=Homo sapiens GN=EMG1 PE=1 SV=4	NEP1_HUMAN	27 kDa	74%	1	4.92%	0.01%	2
Niban-like protein 1 OS=Homo sapiens GN=FAM129B PE=1 SV=2	NIBL1_HUMAN	83 kDa	74%	1	2.73%	0.01%	2
Prefoldin subunit 6 OS=Homo sapiens GN=PFDN6 PE=1 SV=1	PFD6_HUMAN	15 kDa	74%	1	20.20%	0.01%	2
Prostaglandin E synthase 2 OS=Homo sapiens GN=PTGES2 PE=1 SV=1	PGES2_HUMAN	42 kDa	74%	1	6.90%	0.01%	2
Phosphoglycerate kinase 1 OS=Homo sapiens GN=PGK1 PE=1 SV=3	PGK1_HUMAN (+1)	45 kDa	74%	1	3.60%	0.01%	2
Q5VT03|FA22D_HUMAN-R	Q5VT03|FA22D_HUMAN-R	?	74%	1	0.00%	0.01%	2
HAUS augmin-like complex subunit 8 OS=Homo sapiens GN=HAUS8 PE=1 SV=3	Q9BT25|HAUS8_HUMAN	?	74%	1	0.00%	0.01%	2
Ras suppressor protein 1 OS=Homo sapiens GN=RSU1 PE=1 SV=3	RSU1_HUMAN	32 kDa	74%	1	8.66%	0.01%	2
D-3-phosphoglycerate dehydrogenase OS=Homo sapiens GN=PHGDH PE=1 SV=4	SERA_HUMAN	57 kDa	74%	1	4.13%	0.01%	2
Small nuclear ribonucleoprotein Sm D3 OS=Homo sapiens GN=SNRPD3 PE=1 SV=1	SMD3_HUMAN	14 kDa	74%	1	7.14%	0.01%	2
SWI/SNF-related matrix-associated actin-dependent regulator of chromatin subfamily A co	SMRCD_HUMAN	117 kDa	74%	1	2.63%	0.01%	2
SNARE-associated protein Snapin OS=Homo sapiens GN=SNAPIN PE=1 SV=1	SNAPN_HUMAN	15 kDa	74%	1	7.35%	0.01%	2
SWI/SNF-related matrix-associated actin-dependent regulator of chromatin subfamily B me	SNF5_HUMAN	44 kDa	74%	1	3.38%	0.01%	2
U2-associated protein SR140 OS=Homo sapiens GN=SR140 PE=1 SV=2	SR140_HUMAN	118 kDa	74%	1	1.46%	0.01%	2
Signal recognition particle 9 kDa protein OS=Homo sapiens GN=SRP9 PE=1 SV=2	SRP09_HUMAN	10 kDa	74%	1	12.80%	0.01%	2
Cohesin subunit SA-2 OS=Homo sapiens GN=STAG2 PE=1 SV=3	STAG2_HUMAN	141 kDa	74%	1	2.11%	0.01%	2
Symplekin OS=Homo sapiens GN=SYMPK PE=1 SV=2	SYMPK_HUMAN	141 kDa	74%	1	2.51%	0.01%	2
Transcription elongation regulator 1 OS=Homo sapiens GN=TCERG1 PE=1 SV=2	TCRG1_HUMAN	124 kDa	74%	1	1.18%	0.01%	2
Tumor protein D52 OS=Homo sapiens GN=TPD52 PE=1 SV=1	TPD52_HUMAN	20 kDa	74%	1	16.80%	0.01%	2
Nucleoprotein TPR OS=Homo sapiens GN=TPR PE=1 SV=2	TPR_HUMAN	266 kDa	74%	1	0.77%	0.01%	2
Testis-specific Y-encoded-like protein 1 OS=Homo sapiens GN=TSPYL1 PE=1 SV=3	TSYL1_HUMAN	49 kDa	74%	1	4.58%	0.01%	2
Splicing factor U2AF 35 kDa subunit OS=Homo sapiens GN=U2AF1 PE=1 SV=3	U2AF1_HUMAN	28 kDa	74%	1	7.50%	0.01%	2
WD repeat-containing protein 48 OS=Homo sapiens GN=WDR48 PE=1 SV=1	WDR48_HUMAN	76 kDa	74%	1	3.25%	0.01%	2
Zinc finger protein 3 OS=Homo sapiens GN=ZNF3 PE=1 SV=3	ZNF3_HUMAN	51 kDa	74%	1	3.59%	0.01%	2
ZW10 interactor OS=Homo sapiens GN=ZWINT PE=1 SV=2	ZWINT_HUMAN	31 kDa	74%	1	8.30%	0.01%	2
ATP-binding cassette sub-family F member 2 OS=Homo sapiens GN=ABCF2 PE=1 SV=2	ABCF2_HUMAN	71 kDa	74%	1	2.25%	0.00%	1
Abl interactor 1 OS=Homo sapiens GN=ABI1 PE=1 SV=4	ABI1_HUMAN	55 kDa	74%	1	3.54%	0.00%	1
Ataxin-3 OS=Homo sapiens GN=ATXN3 PE=1 SV=2	ATX3_HUMAN	43 kDa	74%	1	0.00%	0.00%	1
Large proline-rich protein BAT2 OS=Homo sapiens GN=BAT2 PE=1 SV=2	BAT2_HUMAN	229 kDa	74%	1	1.07%	0.00%	1
B-cell CLL/lymphoma 7 protein family member C OS=Homo sapiens GN=BCL7C PE=1 SV=2	BCL7C_HUMAN	23 kDa	74%	1	8.29%	0.00%	1
Calcium homeostasis endoplasmic reticulum protein OS=Homo sapiens GN=CHERP PE=2 SV	CHERP_HUMAN	104 kDa	74%	1	2.73%	0.00%	1
DNA polymerase delta catalytic subunit OS=Homo sapiens GN=POLD1 PE=1 SV=2	DPOD1_HUMAN	124 kDa	74%	1	1.26%	0.00%	1
Ezrin OS=Homo sapiens GN=EZR PE=1 SV=4	EZRI_HUMAN	69 kDa	74%	1	2.90%	0.00%	1
Glutamate-rich WD repeat-containing protein 1 OS=Homo sapiens GN=GRWD1 PE=1 SV=1	GRWD1_HUMAN	49 kDa	74%	1	3.59%	0.00%	1
Heterogeneous nuclear ribonucleoprotein C-like 1 OS=Homo sapiens GN=HNRNPCL1 PE=2	HNRCL_HUMAN (+1)	32 kDa	74%	1	4.10%	0.00%	1
Heterogeneous nuclear ribonucleoprotein L-like OS=Homo sapiens GN=HNRPLL PE=1 SV=1	HNRLL_HUMAN	60 kDa	74%	1	3.14%	0.00%	1
Keratin, type II cytoskeletal 8 OS=Homo sapiens GN=KRT8 PE=1 SV=7	K2C8_HUMAN	54 kDa	74%	1	4.76%	0.00%	1
6-phosphofructokinase, liver type OS=Homo sapiens GN=PFKL PE=1 SV=6	K6PL_HUMAN	85 kDa	74%	1	1.92%	0.00%	1
Kinesin-like protein KIF11 OS=Homo sapiens GN=KIF11 PE=1 SV=2	KIF11_HUMAN	119 kDa	74%	1	1.04%	0.00%	1
MORF4 family-associated protein 1 OS=Homo sapiens GN=MRFAP1 PE=1 SV=1	MOFA1_HUMAN	15 kDa	74%	1	18.90%	0.00%	1
Neurotensin/neuromedin N OS=Homo sapiens GN=NTS PE=2 SV=2	NEUT_HUMAN	20 kDa	74%	1	18.20%	0.00%	1
Nucleolar phosphoprotein p130 OS=Homo sapiens GN=NOLC1 PE=1 SV=2	NOLC1_HUMAN	74 kDa	74%	1	3.29%	0.00%	1
Nuclear pore complex protein Nup160 OS=Homo sapiens GN=NUP160 PE=1 SV=2	NU160_HUMAN	149 kDa	74%	1	1.37%	0.00%	1
P18850|ATF6A_HUMAN-R	P18850|ATF6A_HUMAN-R	?	74%	1	0.00%	0.00%	1
26S protease regulatory subunit S10B OS=Homo sapiens GN=PSMC6 PE=1 SV=1	PRS10_HUMAN	44 kDa	74%	1	4.37%	0.00%	1
26S protease regulatory subunit 6A OS=Homo sapiens GN=PSMC3 PE=1 SV=3	PRS6A_HUMAN	49 kDa	74%	1	4.56%	0.00%	1
Spindle and kinetochore-associated protein 3 OS=Homo sapiens GN=SKA3 PE=1 SV=2	Q8IX90|SKA3_HUMAN	?	74%	1	0.00%	0.00%	1
La-related protein 4B OS=Homo sapiens GN=LARP4B PE=1 SV=3	Q92615|LAR4B_HUMAN	?	74%	1	0.00%	0.00%	1
Biorientation of chromosomes in cell division protein 1 OS=Homo sapiens GN=BOD1 PE=1 S	Q96IK1|BOD1_HUMAN	?	74%	1	0.00%	0.00%	1
RNA-binding protein 7 OS=Homo sapiens GN=RBM7 PE=1 SV=1	RBM7_HUMAN	31 kDa	74%	1	6.77%	0.00%	1
40S ribosomal protein S5 OS=Homo sapiens GN=RPS5 PE=1 SV=4	RS5_HUMAN	23 kDa	74%	1	7.35%	0.00%	1
SFRS2-interacting protein OS=Homo sapiens GN=SFRS2IP PE=1 SV=1	SFRIP_HUMAN	129 kDa	74%	1	2.53%	0.00%	1
Leucine-rich repeat protein SHOC-2 OS=Homo sapiens GN=SHOC2 PE=1 SV=2	SHOC2_HUMAN	65 kDa	74%	1	4.64%	0.00%	1
Transcription factor Sp4 OS=Homo sapiens GN=SP4 PE=1 SV=1	SP4_HUMAN	82 kDa	74%	1	3.44%	0.00%	1
General transcription factor 3C polypeptide 4 OS=Homo sapiens GN=GTF3C4 PE=1 SV=2	TF3C4_HUMAN	92 kDa	74%	1	2.31%	0.00%	1
DNA topoisomerase 2-alpha OS=Homo sapiens GN=TOP2A PE=1 SV=3	TOP2A_HUMAN	174 kDa	74%	1	1.05%	0.00%	1
TRM1-like protein OS=Homo sapiens GN=TRM1L PE=1 SV=2	TRM1L_HUMAN	82 kDa	74%	1	3.55%	0.00%	1
Tubulin polyglutamylase TTLL7 OS=Homo sapiens GN=TTLL7 PE=2 SV=2	TTLL7_HUMAN	103 kDa	74%	1	3.27%	0.00%	1
SUMO-conjugating enzyme UBC9 OS=Homo sapiens GN=UBE2I PE=1 SV=1	UBC9_HUMAN	18 kDa	74%	1	12.00%	0.00%	1
E3 ubiquitin-protein ligase UBR2 OS=Homo sapiens GN=UBR2 PE=1 SV=1	UBR2_HUMAN	201 kDa	74%	1	0.74%	0.00%	1
Ubiquitin carboxyl-terminal hydrolase isozyme L5 OS=Homo sapiens GN=UCHL5 PE=1 SV=3	UCHL5_HUMAN	38 kDa	74%	1	7.29%	0.00%	1
Zinc finger CCHC domain-containing protein 8 OS=Homo sapiens GN=ZCCHC8 PE=1 SV=2	ZCHC8_HUMAN	79 kDa	74%	1	2.69%	0.00%	1
Zinc finger protein 609 OS=Homo sapiens GN=ZNF609 PE=1 SV=2	ZN609_HUMAN	151 kDa	74%	1	1.70%	0.00%	1
Anaphase-promoting complex subunit 4 OS=Homo sapiens GN=ANAPC4 PE=1 SV=2	APC4_HUMAN	92 kDa	73%	1	1.98%	0.02%	4
Cell division cycle-associated protein 2 OS=Homo sapiens GN=CDCA2 PE=1 SV=1	CDCA2_HUMAN	113 kDa	72%	1	0.88%	0.00%	1
DNA repair protein RAD51 homolog 3 OS=Homo sapiens GN=RAD51C PE=1 SV=1	RA51C_HUMAN	42 kDa	72%	1	5.85%	0.00%	1
U4/U6 small nuclear ribonucleoprotein Prp31 OS=Homo sapiens GN=PRPF31 PE=1 SV=2	PRP31_HUMAN	55 kDa	70%	1	4.21%	0.01%	2
Cysteine and glycine-rich protein 1 OS=Homo sapiens GN=CSRP1 PE=1 SV=3	CSRP1_HUMAN	21 kDa	70%	1	11.40%	0.01%	2
Transcriptional adapter 3 OS=Homo sapiens GN=TADA3 PE=1 SV=1	O75528|TADA3_HUMAN	?	69%	1	0.00%	0.01%	3
Protein NEDD1 OS=Homo sapiens GN=NEDD1 PE=2 SV=1	NEDD1_HUMAN	72 kDa	68%	1	2.12%	0.01%	2
M-phase phosphoprotein 6 OS=Homo sapiens GN=MPHOSPH6 PE=1 SV=1	MPH6_HUMAN	19 kDa	67%	1	9.38%	0.00%	1
Q58WW2|DCAF6_HUMAN-R	Q58WW2|DCAF6_HUMAN-R	?	66%	1	0.00%	0.01%	2
Q6ZS81|WDFY4_HUMAN-R	Q6ZS81|WDFY4_HUMAN-R	?	66%	1	0.00%	0.00%	1
CD99 antigen OS=Homo sapiens GN=CD99 PE=1 SV=1	CD99_HUMAN	19 kDa	64%	1	32.40%	0.01%	3
Tubulin gamma-1 chain OS=Homo sapiens GN=TUBG1 PE=1 SV=2	TBG1_HUMAN (+1)	51 kDa	63%	1	6.43%	0.00%	1
Nucleoplasmin-3 OS=Homo sapiens GN=NPM3 PE=1 SV=3	NPM3_HUMAN	19 kDa	57%	1	12.90%	0.01%	2
Ras-related protein Rab-5C OS=Homo sapiens GN=RAB5C PE=1 SV=2	RAB5C_HUMAN	23 kDa	53%	1	11.10%	0.01%	2
Microtubule-associated protein 4 OS=Homo sapiens GN=MAP4 PE=1 SV=2	MAP4_HUMAN	121 kDa	52%	1	1.82%	0.00%	1
Ataxin-1-like OS=Homo sapiens GN=ATXN1L PE=1 SV=1	ATX1L_HUMAN	73 kDa	52%	1	3.77%	0.00%	1
U3 small nucleolar RNA-associated protein 6 homolog OS=Homo sapiens GN=UTP6 PE=1 S\	UTP6_HUMAN	70 kDa	51%	1	3.52%	0.00%	1
Protein sprouty homolog 2 OS=Homo sapiens GN=SPRY2 PE=1 SV=1	SPY2_HUMAN	35 kDa	51%	1	5.71%	0.00%	1
Protein Dok-7 OS=Homo sapiens GN=DOK7 PE=1 SV=1	DOK7_HUMAN	53 kDa	48%	1	3.57%	0.00%	1
Integrin-linked protein kinase OS=Homo sapiens GN=ILK PE=1 SV=2	ILK_HUMAN	51 kDa	43%	1	2.21%	0.00%	1
Protein DDI1 homolog 1 OS=Homo sapiens GN=DDI1 PE=2 SV=1	DDI1_HUMAN (+1)	44 kDa	42%	1	2.53%	0.01%	3
Protein scribble homolog OS=Homo sapiens GN=SCRIB PE=1 SV=4	Q14160|SCRIB_HUMAN	?	42%	1	0.00%	0.01%	2
Q9HAW8|UD110_HUMAN-R	Q9HAW8|UD110_HUMAN-R	?	39%	1	0.00%	0.01%	2
Q9NPG1|FZD3_HUMAN-R	Q9NPG1|FZD3_HUMAN-R	?	39%	1	0.00%	0.00%	1
WD repeat and HMG-box DNA-binding protein 1 OS=Homo sapiens GN=WDHD1 PE=1 SV=1	WDHD1_HUMAN	126 kDa	39%	1	1.86%	0.00%	1
Q5JUK9|GGED1_HUMAN-R	Q5JUK9|GGED1_HUMAN-R	?	38%	1	0.00%	0.02%	4
Sperm-associated antigen 5 OS=Homo sapiens GN=SPAG5 PE=1 SV=2	SPAG5_HUMAN	134 kDa	38%	1	1.59%	0.00%	1
Q6YN16|HSDL2_HUMAN-R	Q6YN16|HSDL2_HUMAN-R	?	37%	1	0.00%	0.00%	1
Makorin-2 OS=Homo sapiens GN=MKRN2 PE=2 SV=2	MKRN2_HUMAN	47 kDa	36%	1	6.73%	0.00%	1
RING finger protein 169 OS=Homo sapiens GN=RNF169 PE=1 SV=2	RN169_HUMAN	77 kDa	35%	1	3.39%	0.00%	1
P82675|RT05_HUMAN-R	P82675|RT05_HUMAN-R	?	34%	1	0.00%	0.00%	1
Ribonuclease inhibitor OS=Homo sapiens GN=RNH1 PE=1 SV=2	RINI_HUMAN	50 kDa	34%	1	2.60%	0.00%	1
DNA replication licensing factor MCM4 OS=Homo sapiens GN=MCM4 PE=1 SV=5	MCM4_HUMAN	97 kDa	33%	1	2.20%	0.00%	1
MORC family CW-type zinc finger protein 4 OS=Homo sapiens GN=MORC4 PE=1 SV=2	MORC4_HUMAN	106 kDa	32%	1	1.81%	0.00%	1
Rho guanine nucleotide exchange factor 5 OS=Homo sapiens GN=ARHGEF5 PE=1 SV=2	ARHG5_HUMAN	177 kDa	31%	1	1.88%	0.01%	2
Dynein light chain roadblock-type 1 OS=Homo sapiens GN=DYNLRB1 PE=1 SV=3	DLRB1_HUMAN (+1)	11 kDa	31%	1	12.50%	0.00%	1
Receptor-type tyrosine-protein phosphatase S OS=Homo sapiens GN=PTPRS PE=1 SV=2	PTPRS_HUMAN	217 kDa	31%	1	1.39%	0.00%	1
Q9HCM7|FBSL_HUMAN-R	Q9HCM7|FBSL_HUMAN-R	?	31%	1	0.00%	0.00%	1
Ubiquitin carboxyl-terminal hydrolase 35 OS=Homo sapiens GN=USP35 PE=1 SV=2	UBP35_HUMAN	113 kDa	30%	1	3.34%	0.00%	1
Ataxin-2-like protein OS=Homo sapiens GN=ATXN2L PE=1 SV=2	ATX2L_HUMAN	113 kDa	30%	1	2.70%	0.00%	1
Proteasome assembly chaperone 3 OS=Homo sapiens GN=PSMG3 PE=1 SV=1	PSMG3_HUMAN	13 kDa	29%	1	10.70%	0.01%	3
Sodium channel protein type 2 subunit alpha OS=Homo sapiens GN=SCN2A PE=1 SV=3	SCN2A_HUMAN	228 kDa	28%	1	1.05%	0.00%	1
Q11128|FUT5_HUMAN-R	Q11128|FUT5_HUMAN-R	?	27%	1	0.00%	0.00%	1
Ubiquitin-conjugating enzyme E2 N OS=Homo sapiens GN=UBE2N PE=1 SV=1	UBE2N_HUMAN	17 kDa	27%	1	9.87%	0.00%	1
Q9UPR6|ZFR2_HUMAN-R	Q9UPR6|ZFR2_HUMAN-R	?	26%	1	0.00%	0.01%	2
Probable phospholipid-transporting ATPase IK OS=Homo sapiens GN=ATP8B3 PE=2 SV=3	AT8B3_HUMAN	148 kDa	26%	1	1.83%	0.00%	1
P62899|RL31_HUMAN-R	P62899|RL31_HUMAN-R	?	25%	1	0.00%	0.01%	3
Q9Y2P0|ZN835_HUMAN-R	Q9Y2P0|ZN835_HUMAN-R	?	25%	1	0.00%	0.01%	3
DNA-directed RNA polymerase II subunit RPB9 OS=Homo sapiens GN=POLR2I PE=1 SV=1	RPB9_HUMAN	15 kDa	25%	1	18.40%	0.00%	1
Q8N0X4|CLYBL_HUMAN-R	Q8N0X4|CLYBL_HUMAN-R	?	24%	1	0.00%	0.01%	3
SPS1/STE20-related protein kinase YSK4 OS=Homo sapiens GN=YSK4 PE=2 SV=1	YSK4_HUMAN	151 kDa	24%	1	2.26%	0.00%	1
Q6B0I6|KDM4D_HUMAN-R	Q6B0I6|KDM4D_HUMAN-R	?	23%	1	0.00%	0.01%	3
Cadherin-23 OS=Homo sapiens GN=CDH23 PE=1 SV=1	CAD23_HUMAN	370 kDa	23%	1	1.01%	0.01%	3
Q8NHP1|ARK74_HUMAN-R	Q8NHP1|ARK74_HUMAN-R	?	23%	1	0.00%	0.01%	2
Tripartite motif-containing protein 4 OS=Homo sapiens GN=TRIM4 PE=2 SV=2	TRIM4_HUMAN	57 kDa	23%	1	2.20%	0.01%	2
FH1/FH2 domain-containing protein 1 OS=Homo sapiens GN=FHOD1 PE=1 SV=3	FHOD1_HUMAN	127 kDa	23%	1	1.63%	0.00%	1
RING finger protein 103 OS=Homo sapiens GN=RNF103 PE=2 SV=1	RN103_HUMAN	79 kDa	23%	1	5.11%	0.00%	1
Uncharacterized protein C10orf84 OS=Homo sapiens GN=C10orf84 PE=2 SV=1	CJ084_HUMAN	27 kDa	23%	1	15.50%	0.00%	1
Transmembrane 7 superfamily member 3 OS=Homo sapiens GN=TM7SF3 PE=2 SV=1	TM7S3_HUMAN	64 kDa	23%	1	6.14%	0.00%	1
Q8NDQ6|ZN540_HUMAN-R	Q8NDQ6|ZN540_HUMAN-R	?	23%	1	0.00%	0.00%	1
Putative ATP-dependent RNA helicase DHX33 OS=Homo sapiens GN=DHX33 PE=1 SV=1	DHX33_HUMAN	79 kDa	23%	1	1.41%	0.00%	1
Q9NZM1|MYOF_HUMAN-R	Q9NZM1|MYOF_HUMAN-R	?	23%	1	0.00%	0.00%	1
Q96PQ7|KLHL5_HUMAN-R	Q96PQ7|KLHL5_HUMAN-R	?	22%	1	0.00%	0.01%	2
Uncharacterized protein C17orf68 OS=Homo sapiens GN=C17orf68 PE=2 SV=1	CQ068_HUMAN	135 kDa	22%	1	2.79%	0.00%	1
P28749|RBL1_HUMAN-R	P28749|RBL1_HUMAN-R	?	22%	1	0.00%	0.00%	1
Ceramide glucosyltransferase OS=Homo sapiens GN=UGCG PE=2 SV=1	CEGT_HUMAN	45 kDa	21%	1	8.63%	0.01%	2
Q9ULD6|PDZD6_HUMAN-R	Q9ULD6|PDZD6_HUMAN-R	?	21%	1	0.00%	0.00%	1
P18564|ITB6_HUMAN-R	P18564|ITB6_HUMAN-R	?	21%	1	0.00%	0.00%	1
Q9BX97|PLVAP_HUMAN-R	Q9BX97|PLVAP_HUMAN-R	?	20%	1	0.00%	0.00%	1
Transmembrane protein 44 OS=Homo sapiens GN=TMEM44 PE=2 SV=1	TMM44_HUMAN	52 kDa	20%	1	7.16%	0.00%	1
Down syndrome cell adhesion molecule OS=Homo sapiens GN=DSCAM PE=2 SV=2	DSCAM_HUMAN	222 kDa	20%	1	0.85%	0.00%	1
Rho guanine nucleotide exchange factor 1 OS=Homo sapiens GN=ARHGEF1 PE=1 SV=2	ARHG1_HUMAN	102 kDa	20%	1	3.95%	0.00%	1
Q6P3W6|NBPFA_HUMAN-R	Q6P3W6|NBPFA_HUMAN-R	?	19%	1	0.00%	0.00%	1
Q8TE57|ATS16_HUMAN-R	Q8TE57|ATS16_HUMAN-R	?	19%	1	0.00%	0.00%	1
P17029|ZKSC1_HUMAN-R	P17029|ZKSC1_HUMAN-R	?	19%	1	0.00%	0.00%	1
Q9BX46|RBM24_HUMAN-R	Q9BX46|RBM24_HUMAN-R	?	19%	1	0.00%	0.00%	1
Q4VNC0|AT135_HUMAN-R	Q4VNC0|AT135_HUMAN-R	?	19%	1	0.00%	0.00%	1
Plasminogen-related protein B OS=Homo sapiens GN=PLGLB1 PE=1 SV=1	PLGB_HUMAN	11 kDa	18%	1	25.00%	0.00%	1
Q9NY15|STAB1_HUMAN-R	Q9NY15|STAB1_HUMAN-R	?	17%	1	0.00%	0.00%	1
Q9H426|RIMS4_HUMAN-R	Q9H426|RIMS4_HUMAN-R	?	17%	1	0.00%	0.00%	1
Q96KK3|KCNS1_HUMAN-R	Q96KK3|KCNS1_HUMAN-R	?	16%	1	0.00%	0.01%	2
Zinc finger protein 408 OS=Homo sapiens GN=ZNF408 PE=1 SV=1	ZN408_HUMAN	78 kDa	16%	1	2.36%	0.00%	1
Tudor domain-containing protein 12 OS=Homo sapiens GN=TDRD12 PE=2 SV=2	TDR12_HUMAN	133 kDa	16%	1	0.77%	0.00%	1
Collagen alpha-1(III) chain OS=Homo sapiens GN=COL3A1 PE=1 SV=4	CO3A1_HUMAN	139 kDa	16%	1	3.82%	0.00%	1
Leucine-rich repeat-containing protein 37B OS=Homo sapiens GN=LRRC37B PE=2 SV=2	LR37B_HUMAN	106 kDa	16%	1	2.64%	0.00%	1
65 kDa Yes-associated protein OS=Homo sapiens GN=YAP1 PE=1 SV=1	YAP1_HUMAN	49 kDa	15%	1	8.37%	0.00%	1
Q01082|SPTB2_HUMAN-R	Q01082|SPTB2_HUMAN-R	?	15%	1	0.00%	0.00%	1
P46821|MAP1B_HUMAN-R	P46821|MAP1B_HUMAN-R	?	15%	1	0.00%	0.00%	1
5'-AMP-activated protein kinase subunit gamma-3 OS=Homo sapiens GN=PRKAG3 PE=2 SV	AAKG3_HUMAN	54 kDa	14%	1	3.68%	0.00%	1
Ankyrin repeat and SOCS box protein 15 OS=Homo sapiens GN=ASB15 PE=2 SV=2	ASB15_HUMAN	66 kDa	14%	1	2.04%	0.00%	1
Thyroid adenoma-associated protein OS=Homo sapiens GN=THADA PE=2 SV=1	THADA_HUMAN	220 kDa	14%	1	1.08%	0.00%	1
Solute carrier family 26 member 6 OS=Homo sapiens GN=SLC26A6 PE=1 SV=1	S26A6_HUMAN	83 kDa	14%	1	4.74%	0.00%	1
Q8N4S0|CCD82_HUMAN-R	Q8N4S0|CCD82_HUMAN-R	?	14%	1	0.00%	0.00%	1
Q9HBG4|VPP4_HUMAN-R	Q9HBG4|VPP4_HUMAN-R	?	14%	1	0.00%	0.00%	1
Fatty acid synthase OS=Homo sapiens GN=FASN PE=1 SV=2	FAS_HUMAN	273 kDa	14%	1	1.51%	0.00%	1
Zinc finger protein 446 OS=Homo sapiens GN=ZNF446 PE=2 SV=1	ZN446_HUMAN	49 kDa	14%	1	7.56%	0.00%	1
Tight junction protein ZO-1 OS=Homo sapiens GN=TJP1 PE=1 SV=3	ZO1_HUMAN	195 kDa	14%	1	2.06%	0.00%	1
McKusick-Kaufman/Bardet-Biedl syndromes putative chaperonin OS=Homo sapiens GN=M	MKKS_HUMAN	62 kDa	13%	1	1.93%	0.00%	1
Neural proliferation differentiation and control protein 1 OS=Homo sapiens GN=NPDC1 PE:	NPDC1_HUMAN	35 kDa	13%	1	11.40%	0.00%	1
Peroxisomal 2,4-dienoyl-CoA reductase OS=Homo sapiens GN=DECR2 PE=1 SV=1	DECR2_HUMAN	31 kDa	13%	1	9.93%	0.00%	1
SOSS complex subunit B1 OS=Homo sapiens GN=OBFC2B PE=1 SV=1	Q9BQ15|SOSB1_HUMAN	?	13%	1	0.00%	0.00%	1
P57729|RAB38_HUMAN-R	P57729|RAB38_HUMAN-R	?	13%	1	0.00%	0.00%	1
